# ﻿Four new species of cave-adapted pseudoscorpions (Pseudoscorpiones, Pseudotyrannochthoniidae) from Guizhou, China

**DOI:** 10.3897/zookeys.1139.96639

**Published:** 2023-01-09

**Authors:** Zhizhong Gao, Yanmeng Hou, Feng Zhang

**Affiliations:** 1 Department of Biology, Xinzhou Teachers University, Xinzhou, Shanxi 034000, China Xinzhou Teachers University Xinzhou China; 2 The Key Laboratory of Zoological Systematics and Application, Institute of Life Science and Green Development, College of Life Sciences, Hebei University, Baoding, Hebei 071002, China Hebei University Baoding China

**Keywords:** *Allochthonius*, *Spelaeochthonius*, taxonomy, troglomorphic

## Abstract

Four new troglomorphic pseudotyrannochthoniid pseudoscorpion species collected from karst caves in Guizhou Province are described with detailed diagnoses and illustrations: *Allochthoniusbainiensis***sp. nov.** from Liangfeng Cave (Xishui County), *Allochthoniuspandus***sp. nov.** from Daozuo Cave (Xishui County), *Allochthoniusxinqiaoensis***sp. nov.** from Sanjie Cave (Fenggang County), and *Spelaeochthoniuswulibeiensis***sp. nov.** from Wulibei Cave (Weining County). *Spelaeochthoniuswulibeiensis***sp. nov.** represents the first record of the genus in China. The diagnostic features of these four new cave-adapted (troglomorphic) species are presented and discussed, as well as compared with closely related species. The data on their distribution, habitat and ecology of the species are also given.

## ﻿Introduction

The genus *Allochthonius* Chamberlin, 1929, belonging to the family Pseudotyrannochthoniidae Beier, 1932, mainly distributed in Asia, lately included two subgenera, *Allochthonius* Chamberlin, 1929 and *Urochthonius* Morikawa, 1954. The subgenus *Urochthonius* has been recently synonymized with *Allochthonius* (Harvey and Harms 2022; WPC 2022). Up to now, the genus *Allochthonius* contains a total of 30 species (nine species from China), and of these 30 species, only nine species have no eyes. Of these nine blind species, only *A.brevitus* Hu & Zhang, 2012 comes from China and it is an epigean species, while the other eight species were found in caves in Japan (Morikawa 1956; Hu and Zhang 2011, 2012; Zhang and Zhang 2014; Gao et al. 2016; Schwarze et al. 2021; Viana and Ferreira 2021; WPC 2022).

The genus *Spelaeochthonius* Morikawa, 1954, belonging to the family Pseudotyrannochthoniidae, was erected by Morikawa (1954). All nine species from this genus (six species from Japan and three species from Korea) were found in caves and are completely eyeless and highly troglomorphic. In general, *Spelaeochthonius* species can be distinguished from other genera in the family by the number of carapaceal setae; the number, shape, and arrangement of the coxal spines, and the shape of the intercoxal tubercle; see Morikawa (1956) and You et al. (2022) for details. *Spelaeochthoniuswulibeiensis* sp. nov. represents the first record of the genus in China, even though it is not characterized by the typical distally pinnate or serrate coxal spines.

Southwest China is one of China’s seven physical geographical regions, including Sichuan, Guizhou and Yunnan Province, Chongqing Municipality, and Xizang Autonomous Region (Tibet). It is also the main distribution area of karst landforms, covering an area of 426,240 km^2^ (Zhang et al. 2001). Guizhou, located in the hinterland of southwest China, is the province with the most widely distributed karst landforms (10.91×10^4^ km^2^, accounting for 61.92% of the total land area of the province) and contains tens of thousands of karst caves that host a large amount of unique and undescribed fauna (Lin 2001). Among the at least 742 cave-dwelling species identified in China, nearly 20% of them are found in Guizhou (Latella 2019). One of the representative groups of cave-dwelling arthropods is subterranean-adapted pseudoscorpions. They are usually eyeless and have a hypopigmented body cuticle and elongated body appendages. To date, 54 cave-dwelling pseudoscorpion species from four families (Atemnidae, Chernetidae, Chthoniidae, Neobisiidae) have been described from China (Schawaller 1995; Mahnert 2003, 2009; Mahnert and Li 2016; Gao et al. 2017, 2018, 2020; Li et al. 2017, 2019; Feng et al. 2019, 2020; Zhang et al. 2020; Li and Wang 2021; Hou et al. 2022a, b; Li 2022; Xu et al. 2022), including 12 species from Guizhou. No cavernicolous pseudotyrannochthoniid species have been reported from China yet.

In this study, four new pseudotyrannochthoniid species are presented with detailed diagnoses, descriptions, and illustrations, all of which were collected from caves in Guizhou over the past few years.

## ﻿Materials and methods

As none of these caves in the present study were subject to previous studies or exploration efforts, cave maps are not available. Information on the length of the cave, their temperature and humidity, and the height and width of the cave entrance are provided by using a temperature and humidity meter (LUGE L92-1) and a rangefinder (LEICA X3).

**Figure 1.**
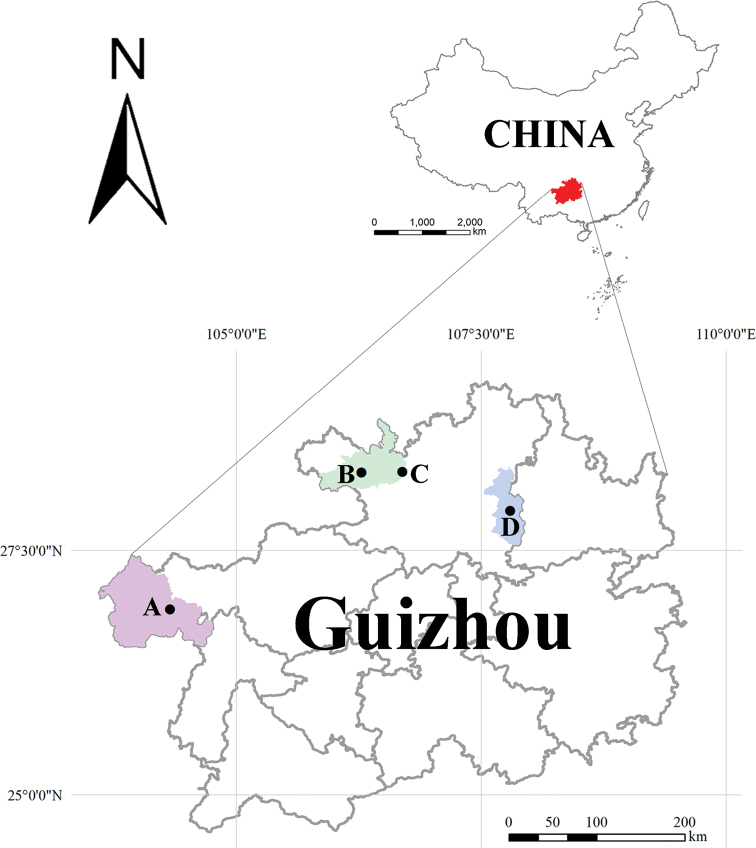
Study area with indication of cave locations representing the type localities. Each color represents an administrative region (red: Guizhou Province; purple: Weining County; green: Xishui County; blue: Fenggang County) **A** Wulibei Cave (*Spelaeochthoniuswulibeiensis* sp. nov.) **B** Liangfeng Cave (*Allochthoniusbainiensis* sp. nov.) **C** Daozuo Cave (*A.pandus* sp. nov.) **D** Sanjie Cave (*A.xinqiaoensis* sp. nov.).

The specimens examined for this study are preserved in 75% alcohol and deposited in the Museum of Hebei University (**MHBU**) (Baoding, China) and the Museum of Southwest University (**MSWU**) (Chongqing, China). Photographs, drawings and measurements were taken using a Leica M205A stereo-microscope equipped with a Leica DFC550 camera and the Inkscape software (Ver. 1.0.2.0). Detailed examination was carried out with an Olympus BX53 general optical microscope. Distribution map was made using ArcGIS 10.6 (Fig. 1). All images were edited and formatted using Adobe Photoshop 2022.

Terminology and measurements follow Chamberlin (1931) with some minor modifications to the terminology of trichobothria (Harvey 1992; Judson 2007) and chelicera (Judson 2007). The chela and legs are measured in lateral view and others are taken in dorsal view. All measurements are given in mm unless noted otherwise. Proportions and measurements of chelicerae, carapace and pedipalps correspond to length/breadth, and those of legs to length/depth. For abbreviations of trichobothria, see Chamberlin (1931).

## ﻿Taxonomy

### ﻿Family Pseudotyrannochthoniidae Beier, 1932

#### ﻿Genus Allochthonius

Taxon classificationAnimaliaPseudoscorpionesPseudotyrannochthoniidae

Chamberlin, 1929

F0146FAC-DC61-53C7-B94A-EB39A66ACC9A

##### Type species.

*Chthoniusopticus* Ellingsen, 1907, by original designation.

#### ﻿ Allochthonius bainiensis sp. nov.

Taxon classificationAnimaliaPseudoscorpionesPseudotyrannochthoniidae

C2A9D751-DEDC-58EF-A3B0-3B61D48DAA07

https://zoobank.org/F80B7B0C-B722-4DD8-8170-2A945BD4698A

**Figure 2.**
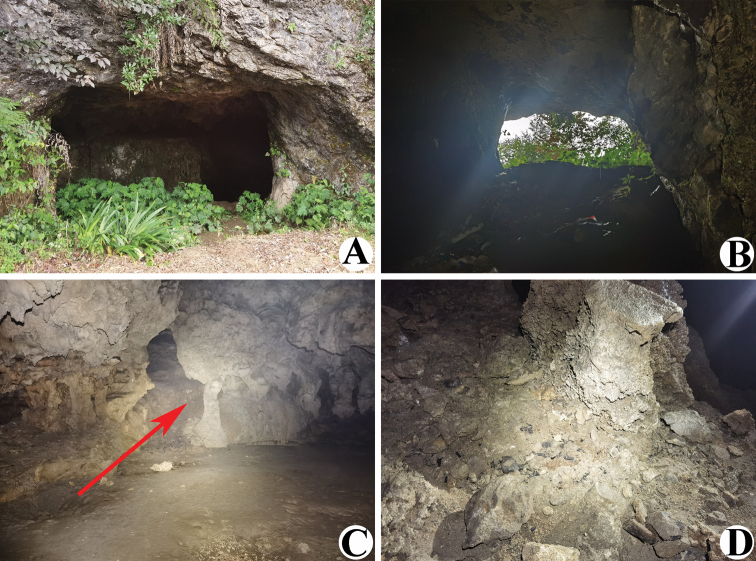
Liangfeng Cave, type locality of *Allochthoniusbainiensis* sp. nov. **A** entrance **B** inside the cave entrance **C, D** area where *A.bainiensis* sp. nov. specimen was collected (red arrow).

**Figure 3.**
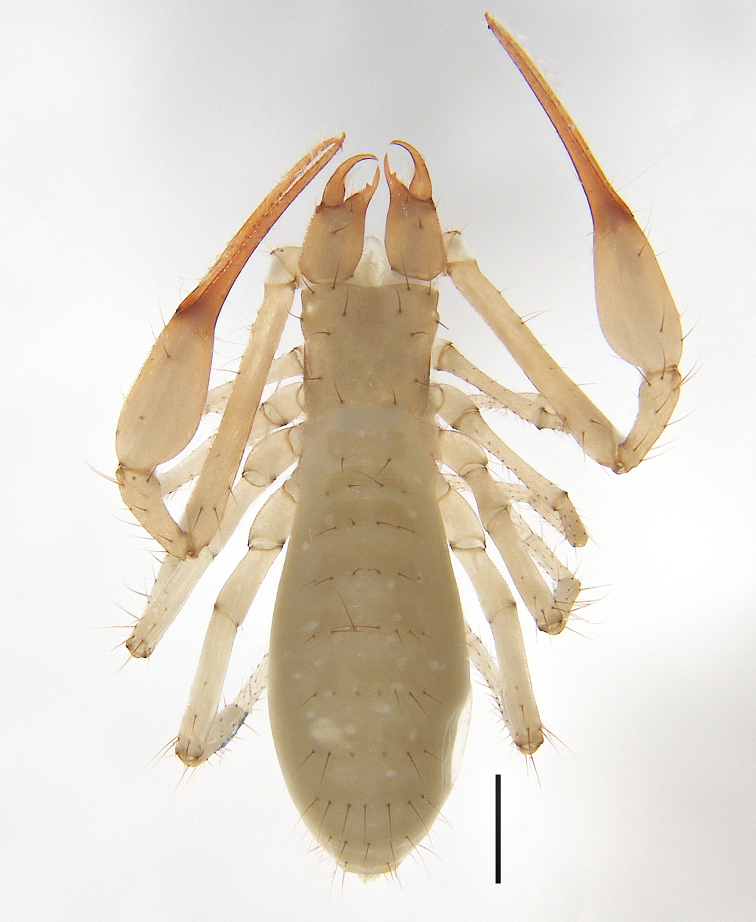
*Allochthoniusbainiensis* sp. nov., holotype female, habitus (dorsal view). Scale bar: 0.50 mm.

**Figure 4.**
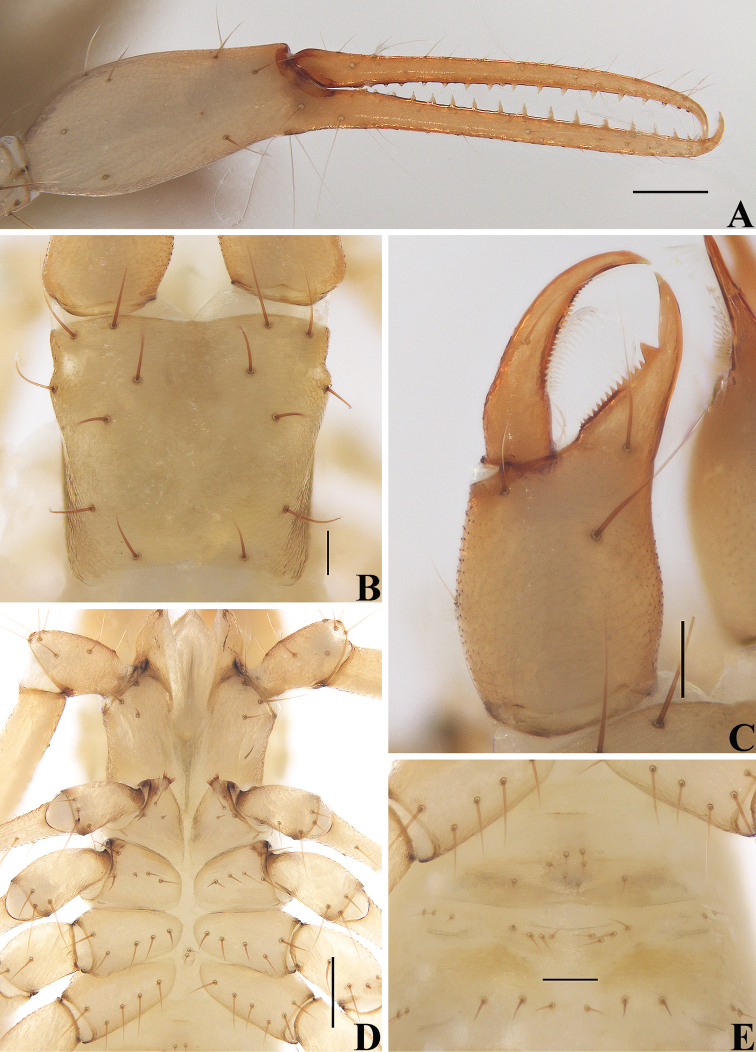
*Allochthoniusbainiensis* sp. nov., holotype female **A** left chela (lateral view) **B** carapace (dorsal view) **C** left chelicera (dorsal view) **D** coxae (ventral view) **E** female genital area (ventral view). Scale bars: 0.20 mm (**A, D**); 0.10 mm (**B, C, E**).

**Figure 5.**
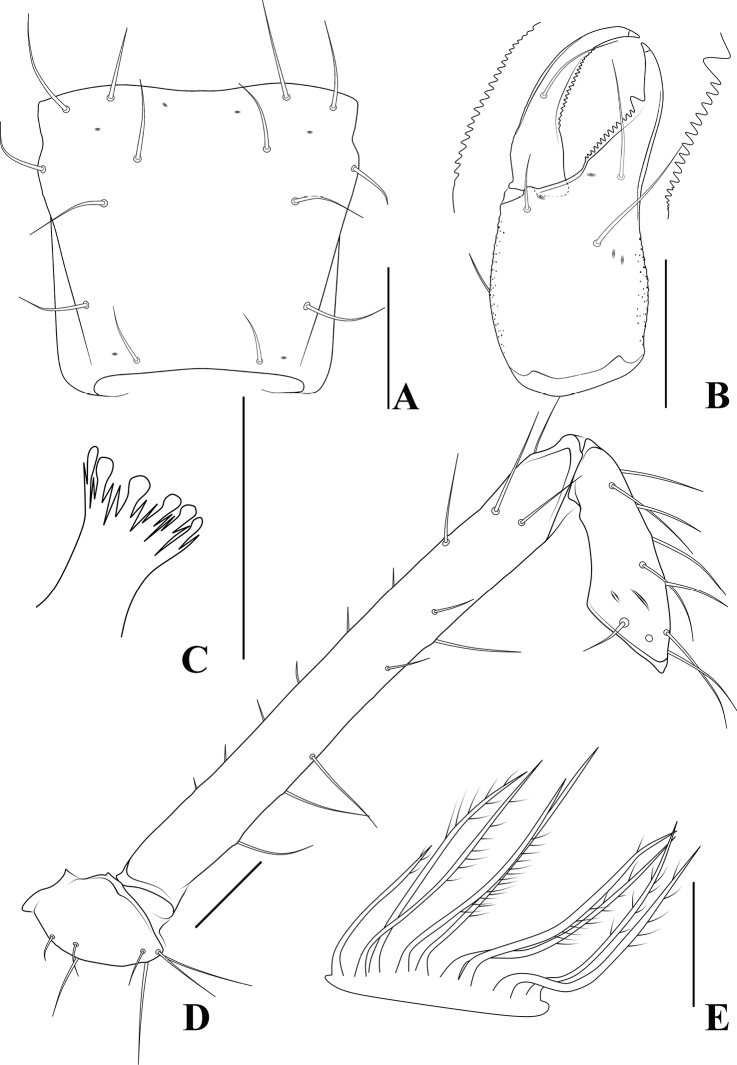
*Allochthoniusbainiensis* sp. nov., holotype female **A** carapace (dorsal view) **B** left chelicera (dorsal view), with details of teeth **C** coxal spines on coxae I (ventral view) **D** left pedipalp (minus chela, dorsal view) **E** rallum. Scale bars: 0.20 mm (**A, B, D**); 0.10 mm (**C, E**).

**Figure 6.**
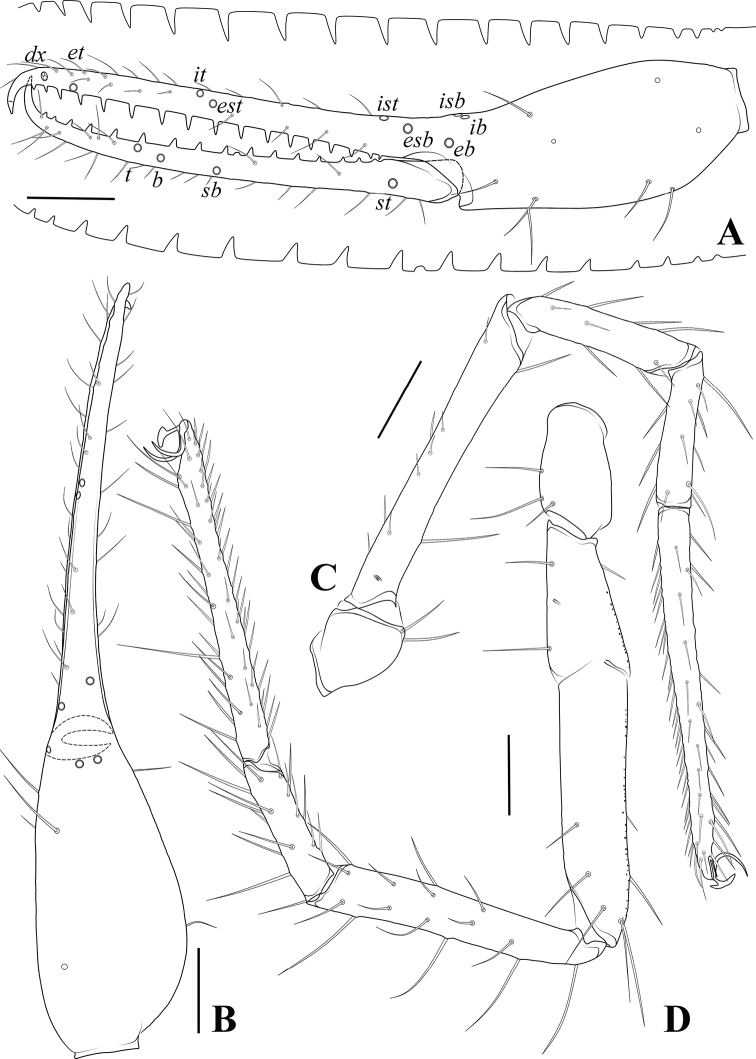
*Allochthoniusbainiensis* sp. nov., holotype female **A** left chela (lateral view), with details of teeth and trichobothrial pattern **B** left chela (dorsal view) **C** leg I (lateral view) **D** leg IV (lateral view). Scale bars: 0.20 mm.

Figs 1B , 2 , 3 , 4 , 5 , 6 Chinese name 白坭异伪蝎

##### Type material.

***Holotype***: China • ♀; Guizhou Province, Xishui County, Donghuang Town, Baini Village, Liangfeng Cave; 28°17.72'N, 106°16.80'E; 1308 m a.s.l., 24 Jul. 2022; Yanmeng Hou, Lu Zhang, Jianzhou Sun and Wenlong Fan leg.; under a stone in the deep zone; Ps.-MHBU-HBUARA#2022-478 (Figs 1B, 2).

##### Diagnosis

**(♀).** The new species can be recognized by the following combination of characters: carapace without eyes or eyespots, posterior margin with two setae, chaetotaxy of carapace: 4–4–2–2–2, 14; cheliceral palm with four setae only; rallum with nine blades (each with fine pinnate, the basal-most blade shorter than the others); coxa I with six coxal spines (tridentate blades, each blade with a central fan-shaped spine terminally) on a tubercle; pedipalps slender, femur 9.07, chela 5.41× longer than broad, both chelal fingers with a row of teeth (fixed chelal finger with 19 teeth; movable chelal finger with 17 teeth), slightly retrorse and pointed.

##### Etymology.

Named after the village of Baini, near the type locality.

##### Description.

**Adult female** (male unknown) (Figs 3–6). ***Color*** (Figs 3, 4): generally pale yellow, chelicerae, pedipalps and tergites slightly darker, soft parts pale. ***Cephalothorax*** (Figs 4B, D, 5A, C): carapace subquadrate, 0.87× longer than broad, gently narrowed posteriorly; surface smooth, without furrows but with six lyrifissures and the posterior part with squamous sculpturing; no traces of eyes; epistomal process absent, space between median setae slightly recurved; with 14 setae arranged 4: 4: 2: 2: 2, preocular setae absent, most setae heavy, long and gently curved. Chaetotaxy of coxae: P 3, I 4, II 5, III 5, IV 5–6; manducatory process with two acuminate distal setae, anterior seta less than 1/2 length of medial seta; coxal spines present on coxa I only, consisting of a tubercle expanded terminally into a characteristic “spray” or “fan” of six elevated processes which extend apically, subequal in length (Figs 4D, 5C); bisetose intercoxal tubercle present between coxae III and IV (Fig. 4D). ***Chelicera*** (Figs 4C, 5B, E): large, approximately as long as carapace, 2.37× longer than broad; four setae present on hand, all setae acuminate, ventrobasal seta shorter than others; movable finger with a medial seta; exterior condylar lyrifissure and exterior lyrifissure exist, palm with two extra setae (close to sub-basal seta). Cheliceral palm with moderate hispid granulation on both ventral and dorsal sides. Both fingers well provided with teeth, fixed finger with 14 acute teeth, distal one largest, plus five small basal teeth, 19 in total; movable finger with 21 retrorse contiguous teeth of equal length; galea absent. Serrula exterior with 18 blades and serrula interior with 12 blades. Rallum in two rows and composed of nine blades with fine pinnate, of which the basal-most blade shorter than the others (Fig. 5E). ***Pedipalp*** (Figs 4A, 5D, 6A, B): long and slender, trochanter 1.68, femur 9.07, patella 3.06, chela 5.41, hand 2.29× longer than broad; femur 2.62× longer than patella; movable chelal finger 1.44× longer than hand and 0.61× longer than chela. Setae generally long and acuminate; two distal lyrifissures present on patella (Fig. 5D). Chelal palm robust and slightly constricted towards fingers. Fixed chelal finger and hand with eight trichobothria, movable chelal finger with four trichobothria, *ib*, *isb*, *eb*, *esb*, and *ist* clustered at the base of fixed finger, *ist* slightly distal to *esb*; *it* slightly distal to *est*, situated subdistally; *et* situated subdistally, very close to chelal teeth; *dx* situated distal to *et*, near the tip of fixed finger; *sb* situated closer to *b* than to *st* (Fig. 6A). Microsetae (chemosensory setae) absent on hand and both palpal fingers. Sensilla absent. Both chelal fingers with a row of teeth, homodentate, spaced regularly along the margin, larger and well-spaced teeth present in the middle of the row, becoming smaller and closer distally and proximally: fixed chelal finger with 19 teeth, slightly retrorse and pointed; movable chelal finger with 17 teeth (slightly smaller than teeth on fixed chelal finger) and a tubercle between the ninth and tenth teeth (Fig. 6A). Chelal fingers slightly curved in dorsal view (Fig. 6B). ***Opisthosoma***: generally typical, pleural membrane finely granulated. Tergites and sternites undivided; setae uniseriate and acuminate. Tergal chaetotaxy I–XII: 2: 4: 4: 6: 6: 7: 7: 6: 7: 5: TT: 0; tergites IX and X each with an unpaired median seta. Sternal chaetotaxy IV–XII: 10: 11: 11: 11: 11: 9: 8: 0: 2. Anterior genital operculum with eight setae plus 14 setae on posterior margin, with a pair of lyrifissures present anterolateral and posteriolateral to genital opening, respectively (Fig. 4E). ***Legs*** (Fig. 6C, D): generally typical, long, and slender. Fine granulation present on anterodorsal faces of femur IV and patella IV. Femur of leg I 1.77× longer than patella and with one lyrifissure at the base of femur; tarsus 2.55× longer than tibia. Femoropatella of leg IV 4.86× longer than deep and with one lyrifissure at the base of femur; tibia 6.17× longer than deep; with basal tactile setae on both tarsal segments: basitarsus 4.22× longer than deep (TS = 0.24), telotarsus 12.43× longer than deep and 2.29× longer than basitarsus (TS = 0.31). Setae of leg I (trochanter to tibia) 2: 10: 9: 12, setae of leg IV (trochanter to basitarsus) 3: 3: 7: 15: 17. Arolium slightly shorter than the claws, not divided; claws simple. ***Dimensions of female holotype*** (length/breadth or, in the case of the legs, length/depth in mm): body length 2.72. Pedipalps: trochanter 0.32/0.19, femur 1.36/0.15, patella 0.52/0.17, chela 1.84/0.34, hand 0.78/0.34, movable finger length 1.12. Chelicera 0.64/0.27, movable finger length 0.34. Carapace 0.55/0.63. Leg I: trochanter 0.24/0.18, femur 0.76/0.11, patella 0.43/0.10, tibia 0.33/0.07, tarsus 0.84/0.07. Leg IV: trochanter 0.34/0.18, femoropatella 1.02/0.21, tibia 0.74/0.12, basitarsus 0.38/0.09, telotarsus 0.87/0.07.

##### Remarks.

*Allochthoniusbainiensis* sp. nov. is similar to *A.pandus* sp. nov. and *A.xinqiaoensis* sp. nov. in having the same number of setae on the carapace (14) and chelicera (6), while differs in the absence of a pair of curved chelal fingers (dorsal view) and the presence of lower number of teeth on chelal fingers (19 vs. 31–33 or 23 teeth on the fixed chelal finger and 17 vs. 26–28 or 23 teeth on the movable chelal finger).

*Allochthoniusbainiensis* sp. nov. differs from *A.brevitus* and *A.yoshizawai* Viana & Ferreira, 2021 in the number of setae on the anterior of the carapace (4 vs. 6) and the cheliceral hand (5 vs. 6), and the number of rallum blades (9 vs. 11).

*Allochthoniusbainiensis* sp. nov. can be distinguished from *A.ishikawai* Morikawa, 1954 and all *A.ishikawai* subspecies by the number of setae on the carapace (14 vs. 16 or more), the presence of lower number of rallum blades (9 vs. 10) and larger body size (2.72 vs. 2.38 mm, which is the longest body length of all *A.ishikawai* subspecies, for example, female of *A.ishikawaiuyamadensis*, Morikawa, 1954).

*Allochthoniusbainiensis* sp. nov. can be distinguished from the other species of *Allochthonius* by the absence of any traces of eyes (Morikawa 1954, 1956, 1960; Hu and Zhang 2012; Viana and Ferreira 2021; WPC 2022).

##### Distribution and habitat.

This species is only known from the type locality, Liangfeng Cave (Figs 1B, 2), which is located near a road, 0.6 km southeast of Baini Village (Xishui County). This limestone cave has a medium-sized rectangular entrance (~ 3 m high and 5 m wide) with a large horizontally extending interior space. The interior of the cave is mainly divided into three tunnels, the left tunnel extends ~ 200 m, the middle tunnel extends ~ 500 m, and the right tunnel communicates with the middle tunnel, ~ 100 m in length. Human disturbance in the entrance zone is serious, but the deep zone remains pristine. The specimen was collected under a stone near the wall in the deepest part of the middle tunnel. This space is completely dark, with constant temperature and humidity (temperature ~ 9 °C, humidity ~ 90%).

#### ﻿ Allochthonius pandus sp. nov.

Taxon classificationAnimaliaPseudoscorpionesPseudotyrannochthoniidae

A5514B01-6A2D-5F4C-B83D-B46941CC8861

https://zoobank.org/50DA34BD-CAD3-4A28-AFD6-28078FBF21E1

**Figure 7.**
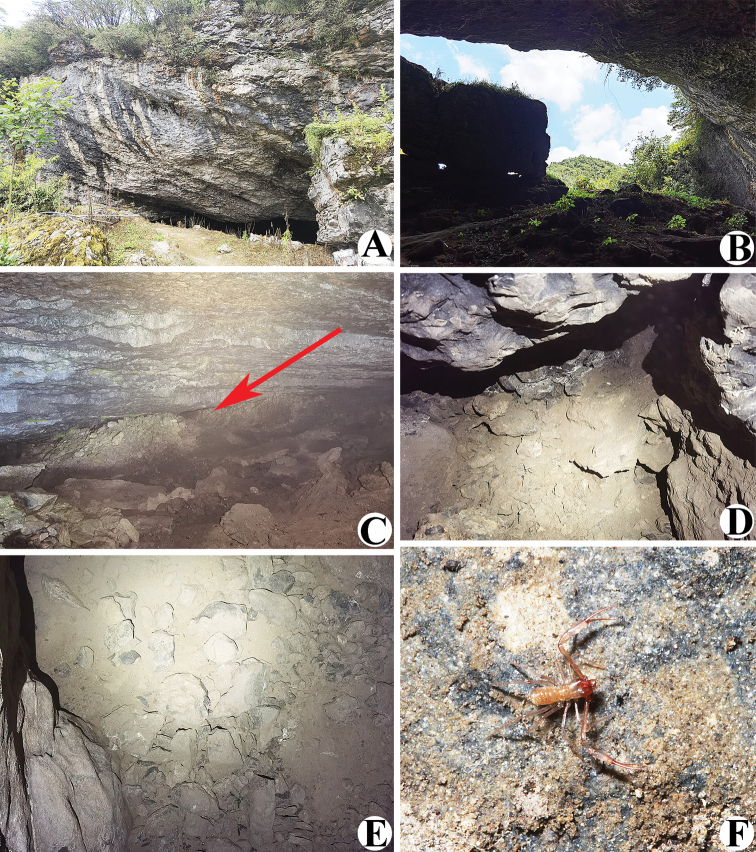
Daozuo Cave, type locality of *Allochthoniuspandus* sp. nov. **A** entrance **B** inside the cave entrance **C** narrow tunnel to the deepest part of the cave (red arrow) **D, E** areas where *A.pandus* sp. nov. specimens were collected **F** live male of *A.pandus* sp. nov. in its natural environment.

**Figure 8.**
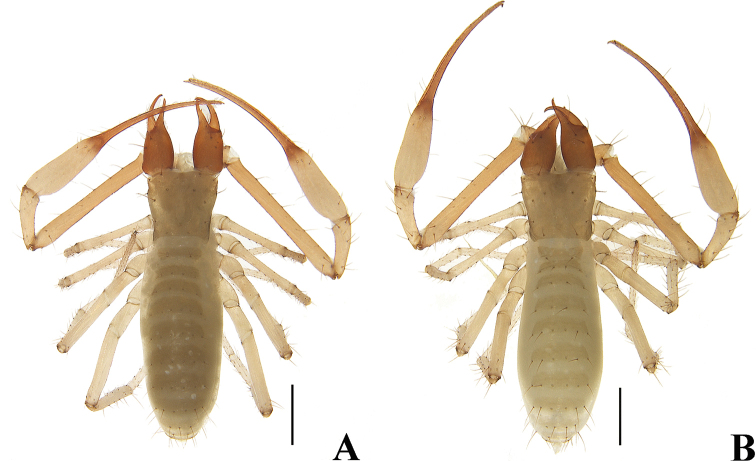
*Allochthoniuspandus* sp. nov. **A** holotype male, habitus (dorsal view) **B** paratype female, habitus (dorsal view). Scale bars: 0.50 mm.

**Figure 9.**
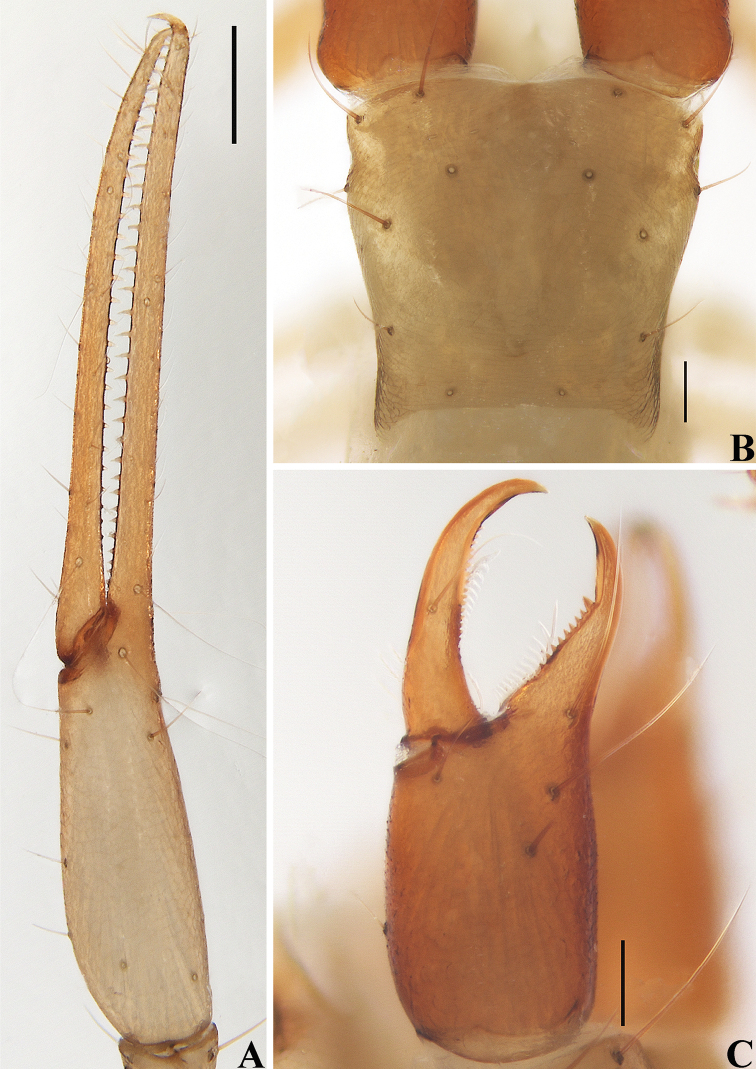
*Allochthoniuspandus* sp. nov., holotype male **A** left chela (lateral view) **B** carapace (dorsal view) **C** left chelicera (dorsal view). Scale bars: 0.20 mm (**A**); 0.10 mm (**B, C**).

**Figure 10.**
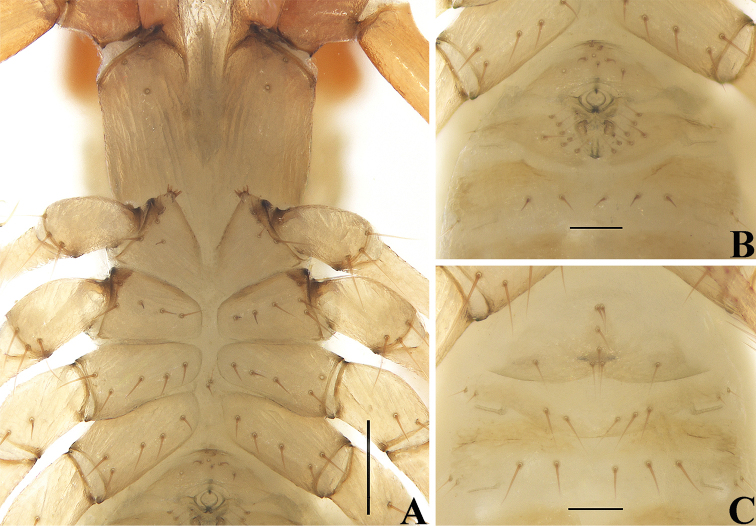
*Allochthoniuspandus* sp. nov., holotype male (**A, B**), paratype female (**C**) **A** coxae (ventral view) **B** male genital area (ventral view) **C** female genital area (ventral view). Scale bars: 0.20 mm (**A**); 0.10 mm (**B, C**).

**Figure 11.**
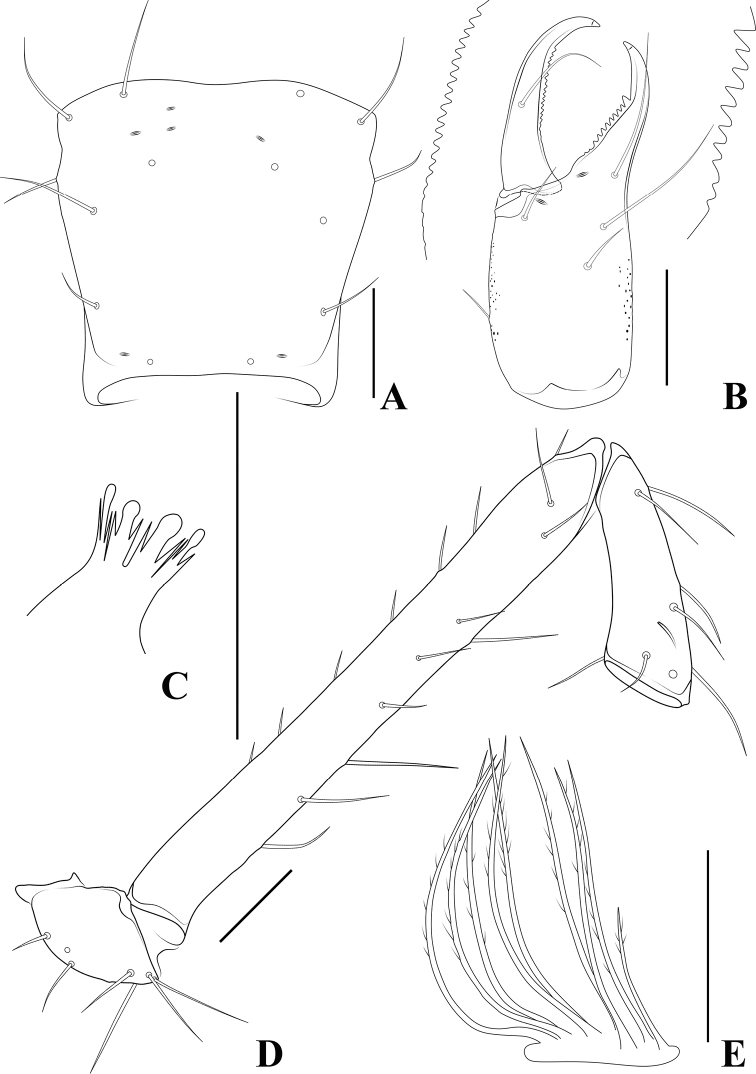
*Allochthoniuspandus* sp. nov., holotype male **A** carapace (dorsal view) **B** left chelicera (dorsal view), with details of teeth **C** coxal spines on coxae I (ventral view) **D** left pedipalp (minus chela, dorsal view) **E** rallum. Scale bars: 0.20 mm (**A, B, D**); 0.10 mm (**C, E**).

**Figure 12.**
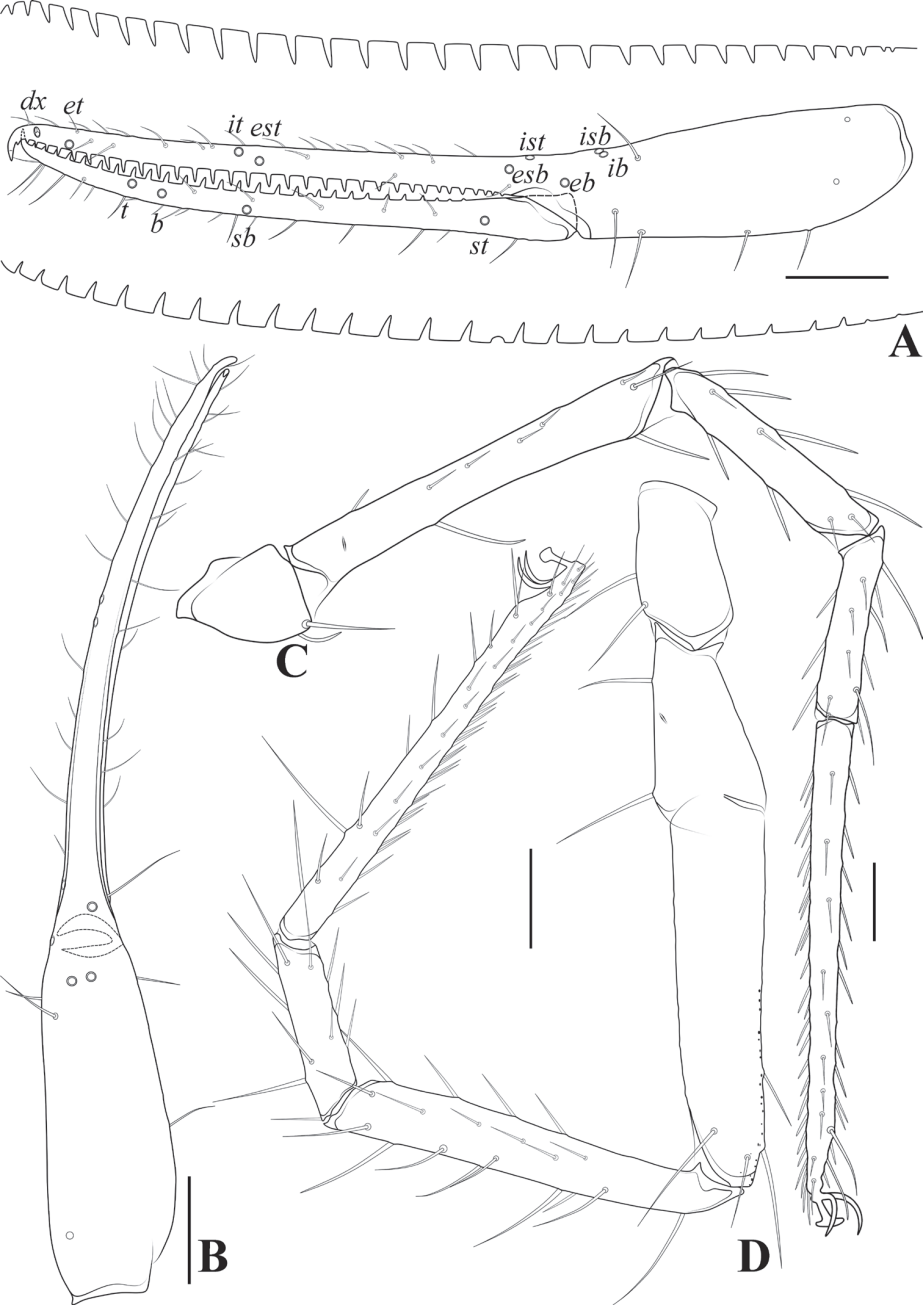
*Allochthoniuspandus* sp. nov., holotype male **A** left chela (lateral view), with details of teeth and trichobothrial pattern **B** left chela (dorsal view) **C** leg I (lateral view) **D** leg IV (lateral view). Scale bars: 0.20 mm.

Figs 1C , 7 , 8 , 9 , 10 , 11 , 12 Chinese name 弯指异伪蝎

##### Type material.

***Holotype***: China • ♂; Guizhou Province, Xishui County, Xianyuan Town, Jinshan Village, Daozuo Cave; 28°18.04'N, 106°41.70'E; 1606 m a.s.l.; 24 Jul. 2022; Yanmeng Hou, Lu Zhang, Jianzhou Sun and Wenlong Fan leg.; under a stone in the deep zone; Ps.-MHBU-HBUARA#2022-47701 (Figs 1C, 7A–E). ***Paratypes***: • 1♂; the same location as the holotype; 28 Aug. 2020; Zegang Feng, Hongru Xu and Yanmeng Hou leg.; Ps.-MHBU-GZXS-20-24 • 2♀; the same data as the holotype; Ps.-MSWU-HBUARA#2022-47702-HBUARA#2022-47703.

##### Diagnosis

**(♂♀).** The new species can be recognized by the following combination of characters: cheliceral palm with five setae; coxa I with four coxal spines (tridentate blades, each blade with a central fan-shaped spine terminally) on a tubercle; pedipalps slender, femur 9.07–10.15 (♂), 8.50–8.60 (♀), chela 7.00–7.52 (♂), 6.64–7.15 (♀) × longer than broad, both chelal fingers with a row of teeth (fixed chelal finger with 31 or 33 teeth; movable chelal finger with 26 or 28 teeth), slightly retrorse and pointed; chela fingers markedly curved in dorsal view.

##### Etymology.

The specific name is derived from the Latin word *pandus* (curved) and refers to the character of the curved chelal fingers.

##### Description.

**Adult males** (Figs 7F, 8A, 9, 10A, B, 11, 12). ***Color*** (Figs 7F, 8A, 9, 10A, B): generally pale yellow, chelicerae, pedipalps and tergites slightly darker, soft parts pale. ***Cephalothorax*** (Figs 9B, 10A, 11A, C): carapace inverted trapezoid, 0.91–0.93× longer than broad, gently narrowed posteriorly; surface smooth, without furrows but with six lyrifissures and the posterior part with squamous sculpturing; no traces of eyes; epistomal process absent, space between median setae slightly recurved; with 14 setae arranged 4: 4: 2: 2: 2, preocular setae absent, most setae heavy, long, and gently curved. Chaetotaxy of coxae: P 3, I 4, II 4–6, III 5, IV 5; manducatory process with two acuminate distal setae, anterior seta less than 1/3 length of medial seta; coxal spines present on coxa I only, consisting of a tubercle expanded terminally into a characteristic “spray” or “fan” of four elevated processes which extend apically, subequal in length (Figs 10A, 11C); bisetose intercoxal tubercle present between coxae III and IV (Fig. 10A). ***Chelicera*** (Figs 9C, 11B, E): large, approximately as long as carapace, 2.56–2.60× longer than broad; five setae and two lyrifissures (exterior condylar lyrifissure and exterior lyrifissure) present on hand, all setae acuminate, ventrobasal seta shorter than others; movable finger with a medial seta. Cheliceral palm with moderate hispid granulation on both ventral and dorsal sides. Both fingers well provided with teeth, fixed finger with ten acute teeth, distal one largest; movable finger with 15 or 16 retrorse contiguous teeth of equal length, plus four or five vestigial, rounded, and contiguous basal teeth, 19–21 in total; galea absent. Serrula exterior with 19 or 20 blades and serrula interior with 10 or 11 blades. Rallum in two rows and composed of nine blades with fine pinnate, of which the basal-most blade shorter than the others (Fig. 11E). ***Pedipalp*** (Figs 9A, 11D, 12A, B): long and slender, trochanter 1.65–1.67, femur 9.07–10.15, patella 3.47–3.57, chela 7.00–7.52, hand 2.60–2.96× longer than broad; femur 2.44–2.64× longer than patella; movable chelal finger 1.59–1.72× longer than hand and 0.62–0.64× longer than chela. Setae generally long and acuminate; one distal lyrifissure present on patella (Fig. 11D). Chelal palm slightly constricted towards fingers. Fixed chelal finger and hand with eight trichobothria, movable chelal finger with four trichobothria, *ib*, *isb*, *eb*, *esb*, and *ist* clustered at the base of fixed finger, *esb* slightly distal to *ist*; *it* slightly distal to *est*, situated subdistally; *et* situated subdistally, very close to chelal teeth; *dx* situated distal to *et*, near the tip of fixed finger; *sb* situated closer to *b* than to *st* (Fig. 12A). Microsetae (chemosensory setae) absent on hand and both palpal fingers. Sensilla absent. Both chelal fingers with a row of teeth, homodentate, spaced regularly along the margin, larger and well-spaced teeth present in the middle of the row, becoming smaller and closer distally and proximally: fixed chelal finger with 31 or 33 teeth, slightly retrorse and pointed; movable chelal finger with 24 or 25 teeth (slightly smaller than teeth on fixed chelal finger), plus two or three vestigial, rounded and contiguous basal teeth, 26–28 in total; a small tubercle between the fourteenth and fifteenth teeth present (Fig. 12A). Chelal fingers markedly curved in dorsal view (Fig. 12B). ***Opisthosoma***: generally typical, pleural membrane finely granulated. Tergites and sternites undivided; setae uniseriate and acuminate. Tergal chaetotaxy I–XII: 2: 4–5: 4–5: 6: 6: 6: 6: 6: 3: 2: TT: 0, tergite IX with an unpaired median seta. Sternal chaetotaxy III–XII: 6–8: 9–10: 9–11: 9: 9–10: 9–10: 7–8: 5–6: 0: 2. Anterior genital operculum with eight setae, genital opening pit-like, with five or six marginal setae on each side, 18–19 in total, with a pair of lyrifissures present anterolateral and posteriolateral to genital opening, respectively (Fig. 10B). ***Legs*** (Fig. 12C, D): generally typical, long, and slender. Fine granulation present on anterodorsal faces of femur IV and patella IV. Femur of leg I 1.64–1.79× longer than patella and with one lyrifissure at the base of femur; tarsus 2.42–2.60× longer than tibia. Femoropatella of leg IV 5.00–5.17× longer than deep and with one lyrifissure at the base of femur; tibia 6.55–7.30× longer than deep; with a long tactile seta on both tarsal segments: basitarsus 4.00–4.86× longer than deep (TS = 0.21–0.25), telotarsus 13.50–14.50× longer than deep and 2.38–2.72× longer than basitarsus (TS = 0.22–0.25). Setae of leg I (trochanter to tibia) 2–3: 9–11: 9–10: 12–13, setae of leg IV (trochanter to basitarsus) 2–3: 2: 4–5: 17–19: 10–11. Arolium slightly shorter than the claws, not divided; claws simple. ***Dimensions of adult males*** (length/breadth or, in the case of the legs, length/depth in mm): body length 1.97–2.23. Pedipalps: trochanter 0.28–0.30/0.17–0.18, femur 1.27–1.32/0.13–0.14, patella 0.50–0.52/0.14–0.15, chela 1.73–1.75/0.23–0.25, hand 0.65–0.68/0.23–0.25, movable chelal finger length 1.08–1.12. Chelicera 0.64–0.65/0.25, movable finger length 0.33–0.34. Carapace 0.53–0.54/0.58. Leg I: trochanter 0.20–0.21/0.15, femur 0.68–0.69/0.08–0.09, patella 0.38–0.42/0.08, tibia 0.30–0.31/0.05–0.06, tarsus 0.75–0.78/0.05–0.06. Leg IV: trochanter 0.29–0.30/0.16–0.17, femoropatella 0.93–0.95/0.18–0.19, tibia 0.72–0.73/0.10–0.11, basitarsus 0.32–0.34/0.07–0.08, telotarsus 0.81–0.87/0.06.

**Adult females** (Figs 8B, 10C). Mostly same as males, but a little larger; chaetotaxy of coxae: P 3, I 4, II 5, III 5, IV 5; tergal chaetotaxy I–XII: 2: 4: 4: 4–6: 6: 6: 6: 6: 4: 2: TT: 0; sternal chaetotaxy IV–XII: 9–10: 10–12: 9: 10–11: 10–12: 7–8: 6: 0: 2; anterior genital operculum with eight or nine setae, posterior margin with nine or ten marginal setae, 17–19 in total; leg IV with a long tactile seta on both tarsal segments: basitarsus 3.78× longer than deep (TS = 0.24), telotarsus 12.86–14.50× longer than deep and 2.56–2.65× longer than basitarsus (TS = 0.20–0.23). Body length 2.10–2.41. Pedipalps: trochanter 0.33/0.19–0.20 (1.65–1.74×), femur 1.36–1.38/0.16 (8.50–8.63×), patella 0.54–0.55/0.18–0.19 (2.89–3.00×), chela 1.86/0.26–0.28 (6.64–7.15×), hand 0.72–0.75/0.26–0.28 (2.68–2.77×), movable chelal finger length 1.16. Chelicera 0.73–0.76/0.26–0.28 (2.71–2.81×), movable finger length 0.39. Carapace 0.55–0.57/0.65 (0.85–0.88×). Leg I: trochanter 0.19/0.17 (1.12×), femur 0.70–0.75/0.10–0.11 (6.82–7.00×), patella 0.43–0.44/0.09 (4.78–4.89×), tibia 0.33–0.35/0.06 (5.50–5.83×), tarsus 0.79–0.84/0.06 (13.17–14.00×). Leg IV: trochanter 0.32–0.34/0.16–0.18 (1.89–2.00×), femoropatella 0.92–1.02/0.19–0.22 (4.64–4.84×), tibia 0.67–0.73/0.10–0.11 (6.64–6.70×), basitarsus 0.34/0.09 (3.78×), telotarsus 0.87–0.90/0.06–0.07 (12.86–14.50×).

##### Remarks.

*Allochthoniuspandus* sp. nov. is similar to *A.xinqiaoensis* sp. nov. in having a pair of distinctly curved chelal fingers and the same chaetotaxy of the carapace (4: 4: 2: 2: 2), but differs by the presence of lower number of blades of coxal spines (4 vs. 6), more rallum blades (9 vs. 8), more slender chela (chela 6.64–7.15 (♀) × vs. 5.44 (♀) × longer than broad), lower number of setae on the coxae (3: 4: 5: 5: 5 vs. 3: 6: 7–9: 5: 5) and more teeth on the chelal fingers (31–33 vs. 23 teeth on the fixed chelal finger and 26–28 vs. 23 teeth on the movable chelal finger); *Allochthoniuspandus* sp. nov. can be distinguished from *A.bainiensis* sp. nov. by the presence of a pair of distinctly curved chelal fingers.

*Allochthoniuspandus* sp. nov. differs from *A.brevitus* and *A.yoshizawai* in the number of setae on the anterior of the carapace (4 vs. 6), the cheliceral hand (5 vs. 6) and tergite II (4–5 vs. 6 or 2), and the number of rallum blades (9 vs. 11) and the presence of more slender pedipalps (e.g., palpal femur 9.07–10.15 (♂) × longer than broad in *A.pandus* sp. nov., while 4.33–4.73 (♂) and 6.50 (♂) × in *A.brevitus* and *A.yoshizawai*, respectively).

*Allochthoniuspandus* sp. nov. can be distinguished from *A.ishikawai* and all *A.ishikawai* subspecies by the number of setae on the carapace (14 vs. 16 or more), the presence of lower number of rallum blades (9 vs. 10) and more teeth on both chelal fingers (26–28 vs. 11–17 teeth on the movable finger and 31–33 vs. 9–17 teeth on the fixed chelal finger).

*Allochthoniuspandus* sp. nov. can be distinguished from the other species of *Allochthonius* by the absence of any traces of eyes (Morikawa 1954, 1956, 1960; Hu and Zhang 2012; Viana and Ferreira 2021; WPC 2022).

##### Distribution and habitat.

This species is only known from the type locality, Daozuo Cave (Figs 1C, 7), which is located near a road, 1 km southwest of Jinshan Village (Xishui County) and is surrounded by rural and agricultural fields. This limestone cave has a large, rectangular entrance (~ 1 m high and 30 m wide) and a total length of ~ 300 m, only a narrow tunnel leads to the deepest part of the cave, which is a slightly wider, low-temperature, high-humidity, and completely lightless environment (temperature ~ 11 °C, humidity > 90%). All specimens were collected under stones in the deepest part of the cave.

#### ﻿ Allochthonius xinqiaoensis sp. nov.

Taxon classificationAnimaliaPseudoscorpionesPseudotyrannochthoniidae

1C08A1E3-D2A0-50C2-8394-CBA818821FC6

https://zoobank.org/F4287D1F-ADBF-47DE-B878-E238375710CD

**Figure 13.**
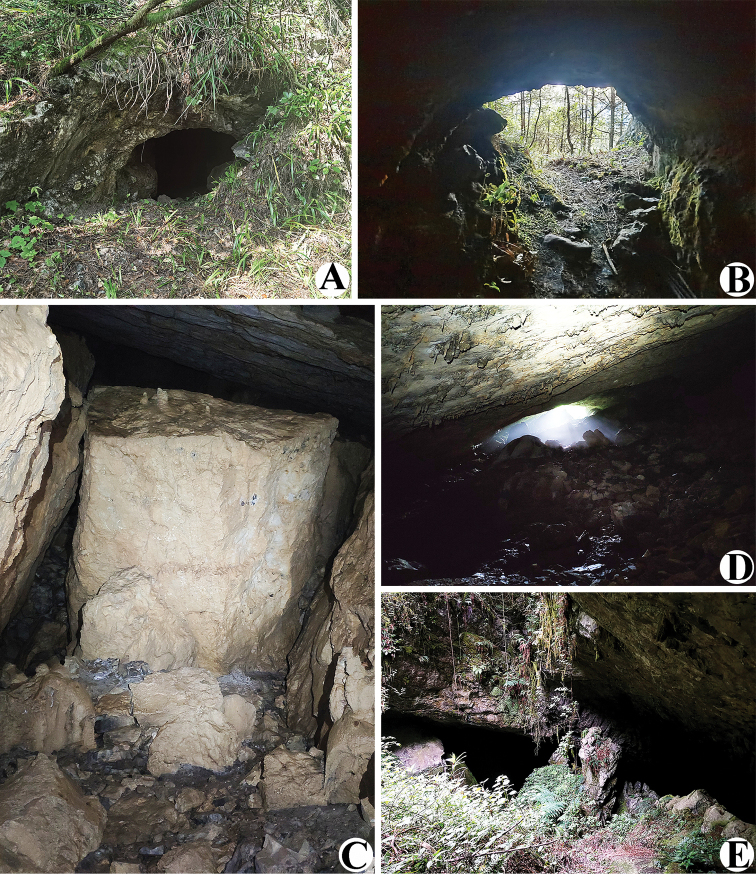
Sanjie Cave, type locality of *Allochthoniusxinqiaoensis* sp. nov. **A** entrance **B** inside the cave entrance **C** area where *A.xinqiaoensis* sp. nov. specimen was collected **D, E** exit.

**Figure 14.**
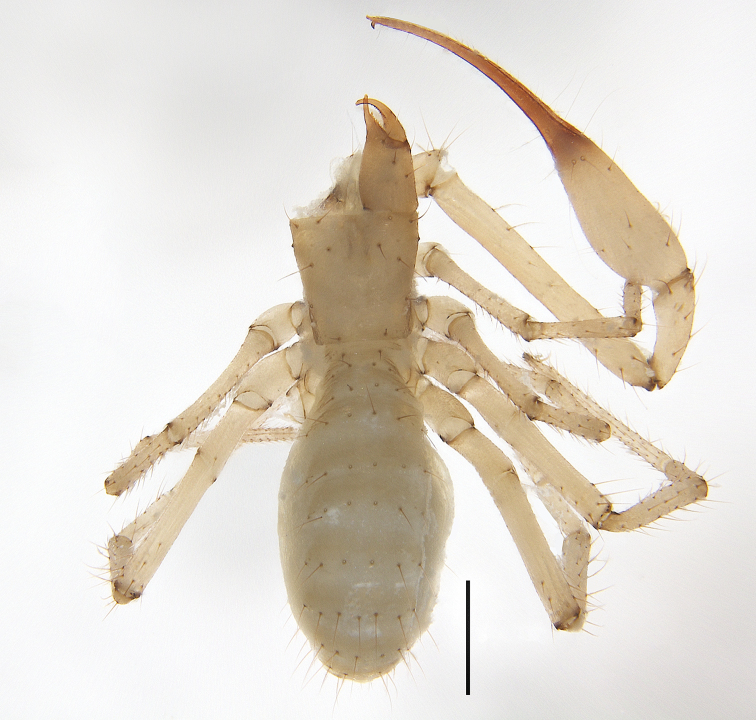
*Allochthoniusxinqiaoensis* sp. nov., holotype female, habitus (minus left chelicera, pedipalp, legs I and IV, dorsal view). Scale bar: 0.50 mm.

**Figure 15.**
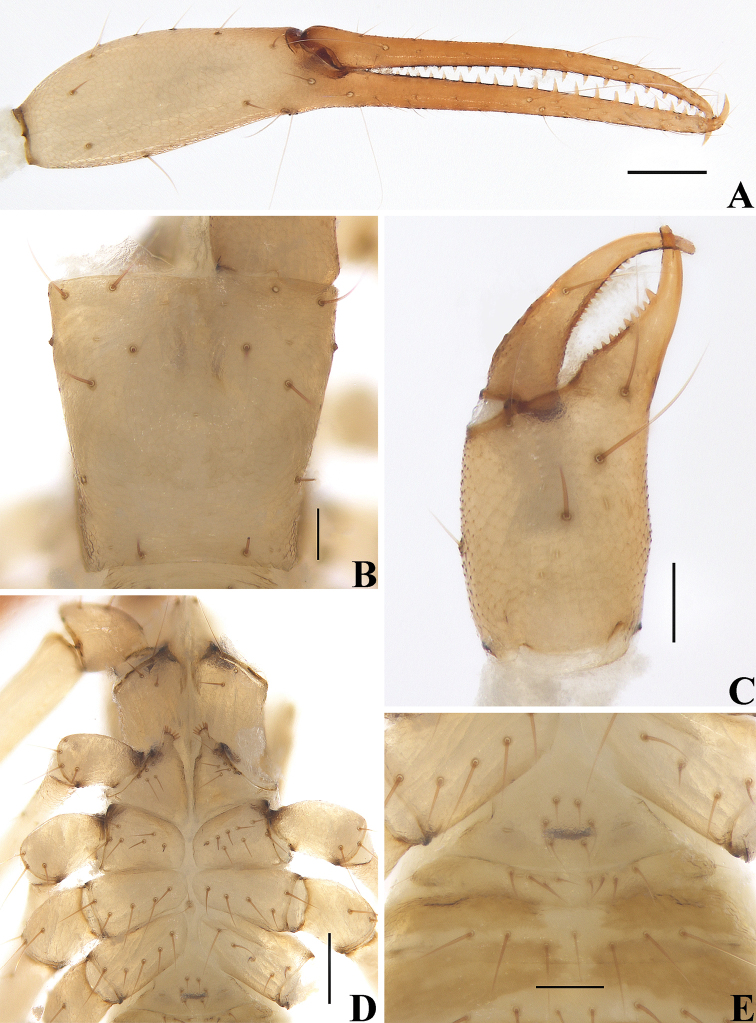
*Allochthoniusxinqiaoensis* sp. nov., holotype female **A** left chela (lateral view) **B** carapace (dorsal view) **C** left chelicera (dorsal view) **D** coxae (ventral view) **E** female genital area (ventral view). Scale bars: 0.20 mm (**A, D**); 0.10 mm (**B, C, E**).

**Figure 16.**
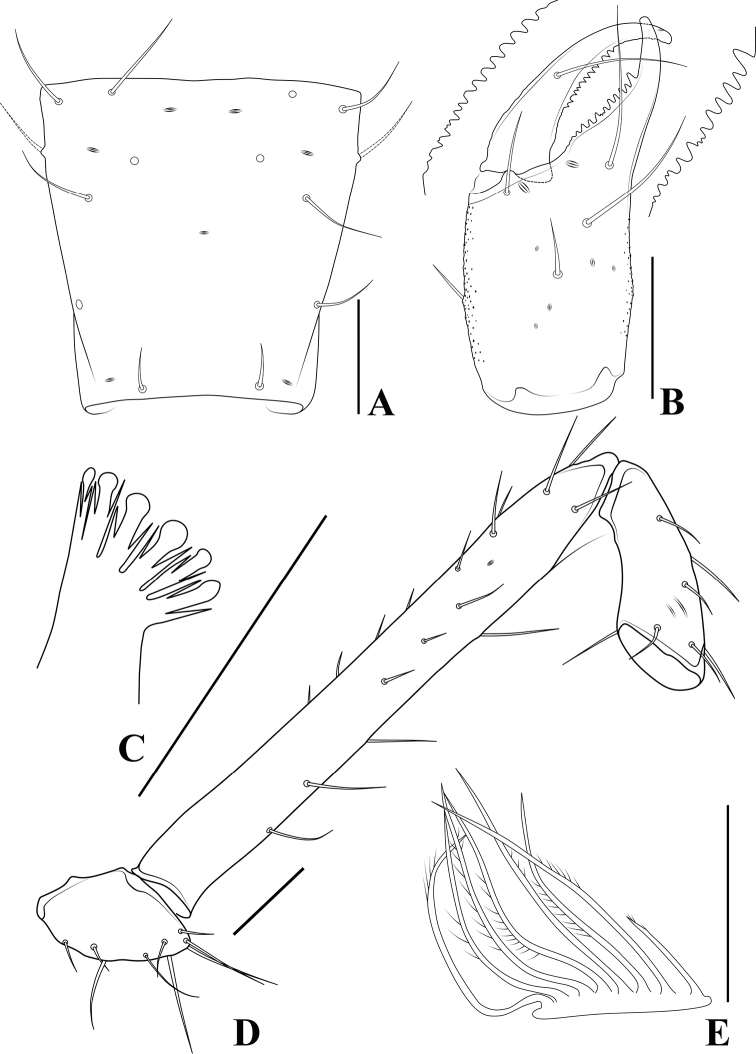
*Allochthoniusxinqiaoensis* sp. nov., holotype female **A** carapace (dorsal view), two broken ocular row setae are shown as dashed lines **B** left chelicera (dorsal view), with details of teeth **C** coxal spines on coxae I (ventral view) **D** left pedipalp (minus chela, dorsal view) **E** rallum. Scale bars: 0.20 mm (**A, B, D**); 0.10 mm (**C, E**).

**Figure 17.**
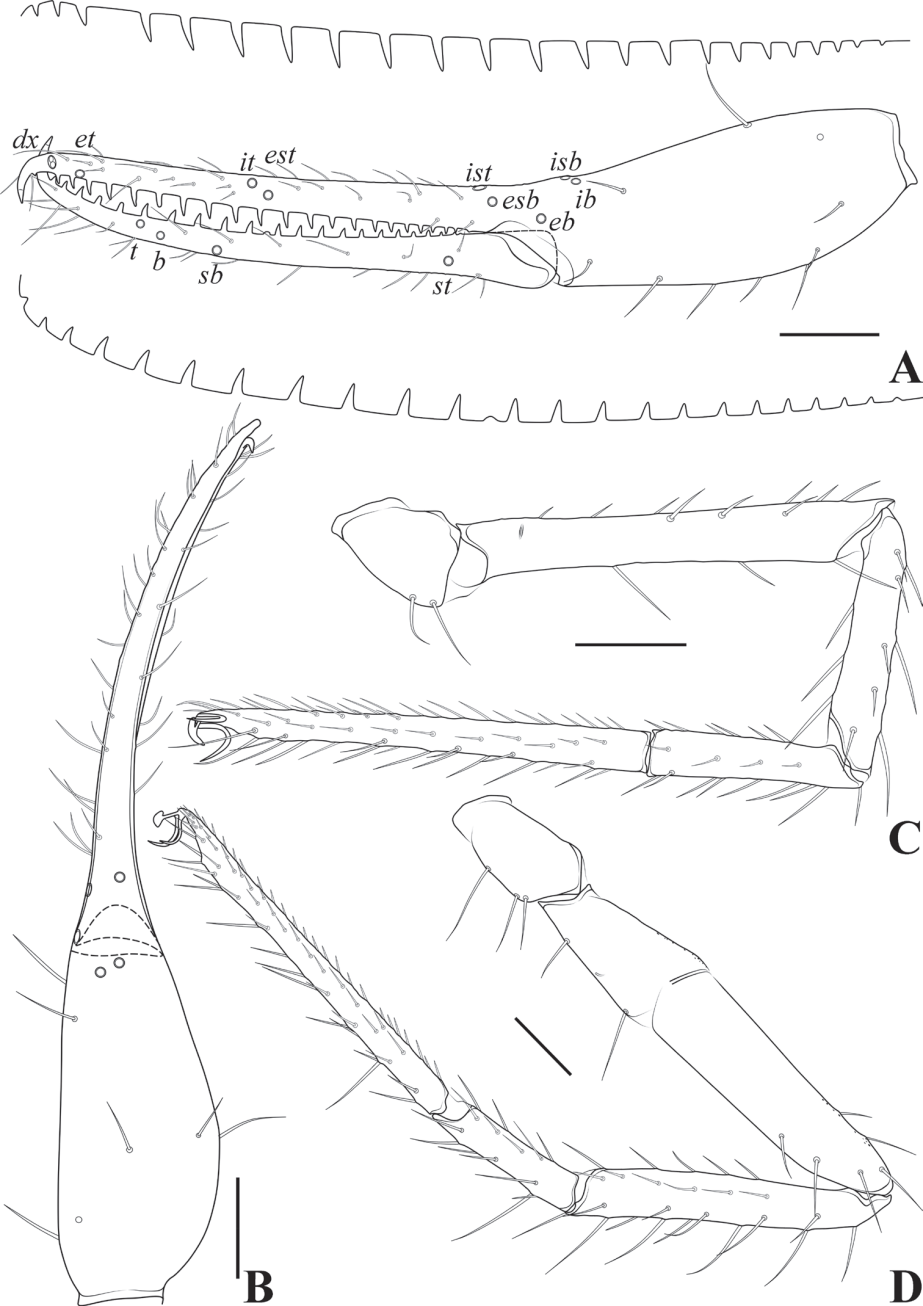
*Allochthoniusxinqiaoensis* sp. nov., holotype female **A** left chela (lateral view), with details of teeth and trichobothrial pattern **B** left chela (dorsal view) **C** leg I (lateral view) **D** leg IV (lateral view). Scale bars: 0.20 mm.

Figs 1D , 13 , 14 , 15 , 16 , 17 Chinese name 新桥异伪蝎

##### Type material.

***Holotype***: China • ♀; Guizhou Province, Fenggang County, Heba Town, Xinqiao Village, Sanjie Cave; 27°54.23'N, 107°47.80'E; 828 m a.s.l.; 26 Jul. 2019; Zegang Feng, Zhaoyi Li and Chen Zhang leg.; under a stone in the deep zone; Ps.-MHBU-GZC190726 (Figs 1D, 13).

##### Diagnosis

**(♀).** The new species can be recognized by the following combination of characters: each cheliceral finger with several small basal teeth between large teeth, most of which appear in pairs, the fingertips blunt, not sharp; rallum with eight blades (each with fine pinnate, the basal-most blade shorter than the others); pedipalps slender, femur 9.71, chela 5.44× longer than broad, both chelal fingers with a row of teeth (each chelal finger with 23 teeth), slightly retrorse and pointed.

##### Etymology.

Named after the village of Xinqiao, near the type locality.

##### Description.

**Adult female** (male unknown) (Figs 14–17). ***Color*** (Figs 14, 15): generally pale yellow, chelicerae, pedipalps and tergites slightly darker, soft parts pale. ***Cephalothorax*** (Figs 15B, D, 16A, C): carapace inverted trapezoid, 1.00× longer than broad, gently narrowed posteriorly; surface smooth, without furrows but with seven lyrifissures and the posterior part with squamous sculpturing; no traces of eyes; epistomal process absent, space between median setae slightly recurved; with 14 setae arranged 4: 4: 2: 2: 2, preocular setae absent, most setae heavy, long and gently curved. Chaetotaxy of coxae: P 3, I 6, II 7–9, III 5, IV 5; manducatory process with two acuminate distal setae, anterior seta less than 1/2 length of medial seta; coxal spines present on coxa I only, consisting of a tubercle expanded terminally into a characteristic “spray” or “fan” of six elevated processes which extend apically, subequal in length (Figs 15D, 16C); bisetose intercoxal tubercle present between coxae III and IV (Fig. 15D). ***Chelicera*** (Figs 15C, 16B, E): large, approximately as long as carapace, 2.38× longer than broad; five setae present on hand, all setae acuminate, ventrobasal seta shorter than others; movable finger with a medial seta; exterior condylar lyrifissure and exterior lyrifissure exist, palm with five extra (surrounding an accessory seta). Cheliceral palm with moderate hispid granulation on both ventral and dorsal sides. Both fingers well provided with teeth, fixed finger with nine acute teeth, distal one largest; movable finger with a slight bump apical tooth and 12 retrorse contiguous teeth of equal length, each finger with several small basal teeth between large teeth, most of which appear in pairs, four on movable finger and six on fixed finger; the fingertips blunt, not sharp; galea represented by a very slight bump on movable finger. Serrula exterior with 17 blades and serrula interior with ten blades. Rallum in two rows and composed of eight blades with fine pinnate, of which the basal-most blade shorter than the others (Fig. 16E). ***Pedipalp*** (Figs 15A, 16D, 17A, B): long and slender, trochanter 1.63, femur 9.71, patella 2.83, chela 5.44, hand 2.25× longer than broad; femur 2.67× longer than patella; movable chelal finger 1.44× longer than hand and 0.60× longer than chela. Setae generally long and acuminate; two distal lyrifissures present on patella, femur with one (Fig. 16D). Chelal palm robust and slightly constricted towards fingers. Fixed chelal finger and hand with eight trichobothria, movable chelal finger with four trichobothria, *ib*, *isb*, *eb*, *esb*, and *ist* clustered at the base of fixed finger, *ist* slightly distal to *esb*; *it* slightly distal to *est*, situated subdistally; *et* situated subdistally, very close to chelal teeth; *dx* situated distal to *et*, near the tip of fixed finger; *sb* situated closer to *b* than to *st* (Fig. 17A). Microsetae (chemosensory setae) absent on hand and both palpal fingers. Sensilla absent. Both chelal fingers with a row of teeth, homodentate, spaced regularly along the margin, larger and well-spaced teeth present in the middle of the row, becoming smaller and closer distally and proximally: fixed chelal finger with 23 teeth, slightly retrorse and pointed; movable chelal finger with 23 teeth (slightly smaller than teeth on fixed chelal finger) and a tubercle between the eleventh and twelfth teeth (Fig. 17A). Chelal fingers markedly curved in dorsal view (Fig. 17B). ***Opisthosoma***: generally typical, pleural membrane finely granulated. Tergites and sternites undivided; setae uniseriate and acuminate. Tergal chaetotaxy I–XII: 3: 4: 4: 6: 6: 6: 6: 7: 5: 4: TT: 0; tergites VIII and IX each with an unpaired median seta; a lyrifissure on each side of tergites I–IX. Sternal chaetotaxy IV–XII: 9: 12: 11: 12: 12: 9: 8: 0: 2. Anterior genital operculum with six setae plus 12 setae on posterior margin, with a pair of lyrifissures present anterolateral and posteriolateral to genital opening, respectively (Fig. 15E). ***Legs*** (Fig. 17C, D): generally typical, long, and slender. Fine granulation present on anterodorsal faces of femur IV and patella IV. Femur of leg I 1.61× longer than patella and with one lyrifissure at the base of femur; tarsus 2.24× longer than tibia. Femoropatella of leg IV 4.74× longer than deep and with one lyrifissure at the base of femur; tibia 6.58× longer than deep; with basal tactile setae on both tarsal segments: basitarsus 4.44× longer than deep (TS = 0.28), telotarsus 14.29× longer than deep and 2.50× longer than basitarsus (TS = 0.20). Setae of leg I (trochanter to tibia) 2: 12: 11: 19, setae of leg IV (trochanter to basitarsus) 3: 2: 6: 24: 14. Arolium slightly shorter than the claws, not divided; claws simple. ***Dimensions of female holotype*** (length/breadth or, in the case of the legs, length/depth in mm): body length 2.01. Pedipalps: trochanter 0.31/0.19, femur 1.36/0.14, patella 0.51/0.18, chela 1.74/0.32, hand 0.72/0.32, movable finger length 1.04. Chelicera 0.57/0.24, movable finger length 0.32. Carapace 0.55/0.55. Leg I: trochanter 0.22/0.17, femur 0.79/0.10, patella 0.49/0.09, tibia 0.38/0.07, tarsus 0.85/0.06. Leg IV: trochanter 0.34/0.19, femoropatella 1.09/0.23, tibia 0.79/0.12, basitarsus 0.40/0.09, telotarsus 1.00/0.07.

##### Remarks.

*Allochthoniusxinqiaoensis* sp. nov. is similar to *A.ishikawaishiragatakiensis* Morikawa, 1954 in having a pair of distinctly curved chelal fingers, but differs by the presence of lower number of rallum blades (8 vs. 10), larger body size (body length 2.01 vs. 1.75 mm) and more chelal fingers teeth (23 vs. 9 on the fixed chelal finger and 23 vs. 11 on the movable chelal finger).

*Allochthoniusxinqiaoensis* sp. nov. can be distinguished from *A.pandus* sp. nov. by the presence of more blades of coxal spines (6 vs. 4), lower number of rallum blades (8 vs. 9), thicker chela (chela 5.44 (♀) × vs. 6.64–7.15 (♀) × longer than broad), more setae on the coxae (3: 6: 7–9: 5: 5 vs. 3: 4: 5: 5: 5) and lower number of teeth on the chelal fingers (23 vs. 31–33 teeth on the fixed chelal finger and 23 vs. 26–28 teeth on the movable chelal finger); *Allochthoniusxinqiaoensis* sp. nov. can be distinguished from *A.bainiensis* sp. nov. by the presence a pair of distinctly curved chelal fingers.

*Allochthoniusxinqiaoensis* sp. nov. differs from *A.brevitus* and *A.yoshizawai* in the number of setae on the anterior of the carapace (4 vs. 6) and the cheliceral hand (5 vs. 6), and the number of rallum blades (8 vs. 11).

*Allochthoniusxinqiaoensis* sp. nov. can be distinguished from *A.ishikawai* and all the other *A.ishikawai* subspecies by the number of setae on the carapace (14 vs. 16 or more), the presence of lower number of rallum blades (8 vs. 10) and more teeth on both chelal fingers (23 vs. 11–17 teeth on the movable chelal finger and 23 vs. 9–17 teeth on the fixed chelal finger).

*Allochthoniusxinqiaoensis* sp. nov. can be distinguished from the other species of *Allochthonius* by the absence of any traces of eyes (Morikawa 1954, 1956, 1960; Hu and Zhang 2012; Viana and Ferreira 2021; WPC 2022).

##### Distribution and habitat.

This species is only known from the type locality, Sanjie Cave (Figs 1D, 13), which is located ~ 1.8 km northeast of Xinqiao Village (Fenggang County). This limestone cave has a small oval entrance (~ 1 m high and 2 m wide), ~ 200 meters in length, with a large, elongated exit at the end of the cave (~ 5 m high and 50 m wide). The interior entirety of the cave is large, inclined and extending downwards. The cave ground was covered with stones. The specimen was collected under a stone ~ 100 m from the cave entrance.

#### ﻿Genus Spelaeochthonius

Taxon classificationAnimaliaPseudoscorpionesPseudotyrannochthoniidae

Morikawa, 1954

B2A039BD-3FE8-5ACE-A5A5-B47AA00A8CC5

##### Type species.

*Spelaeochthoniuskubotai* Morikawa, 1954, by original designation.

#### ﻿ Spelaeochthonius wulibeiensis sp. nov.

Taxon classificationAnimaliaPseudoscorpionesPseudotyrannochthoniidae

B8502145-B46C-58B4-8237-EED883C95229

https://zoobank.org/296D1EC6-9D37-43A4-B971-6330674C6711

**Figure 18.**
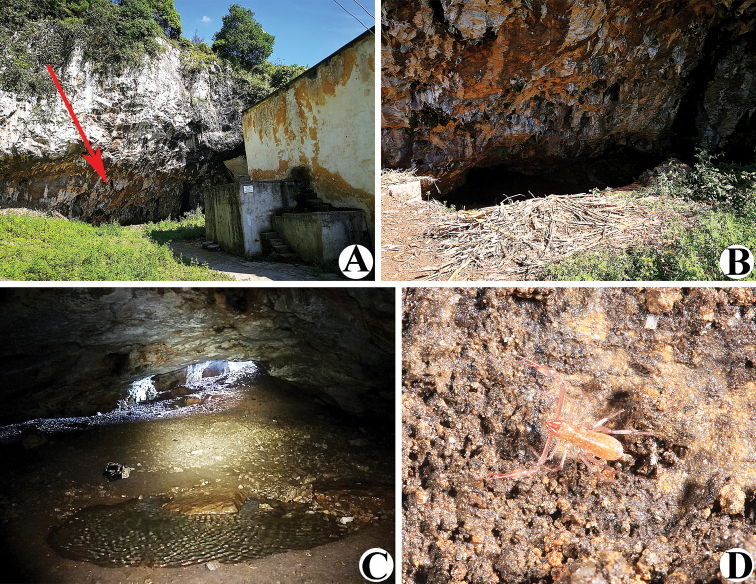
Wulibei Cave, type locality of *Spelaeochthoniuswulibeiensis* sp. nov. **A** cave location (red arrow) **B** entrance **C** inside the cave entrance **D** live male of *S.wulibeiensis* sp. nov. in its natural environment.

**Figure 19.**
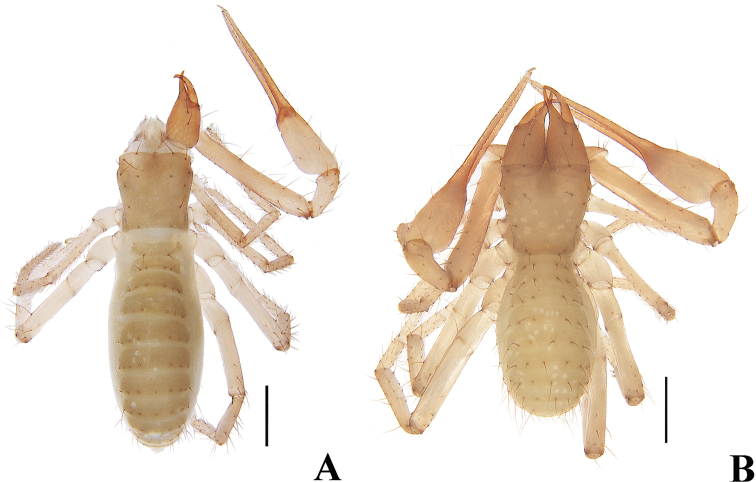
*Spelaeochthoniuswulibeiensis* sp. nov. **A** holotype male, habitus (minus left chelicera, pedipalp, legs I and IV, dorsal view) **B** paratype female, habitus (dorsal view). Scale bars: 0.50 mm.

**Figure 20.**
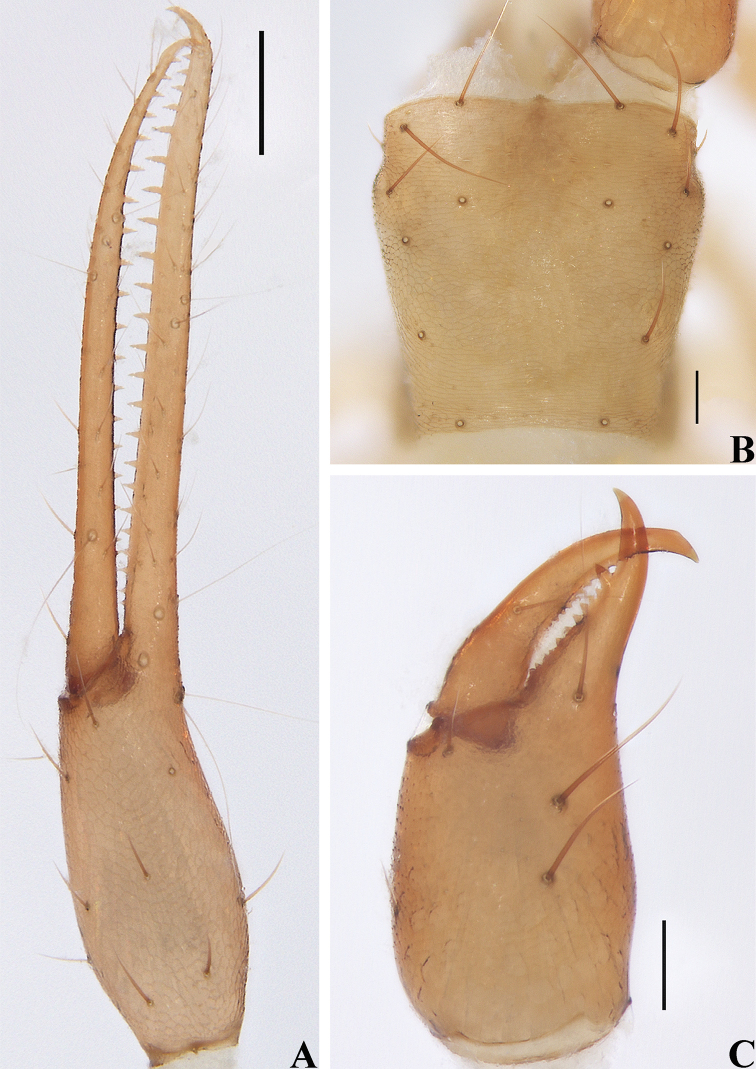
*Spelaeochthoniuswulibeiensis* sp. nov., holotype male **A** left chela (lateral view) **B** carapace (dorsal view) **C** left chelicera (dorsal view). Scale bars: 0.20 mm (**A**); 0.10 mm (**B, C**).

**Figure 21.**
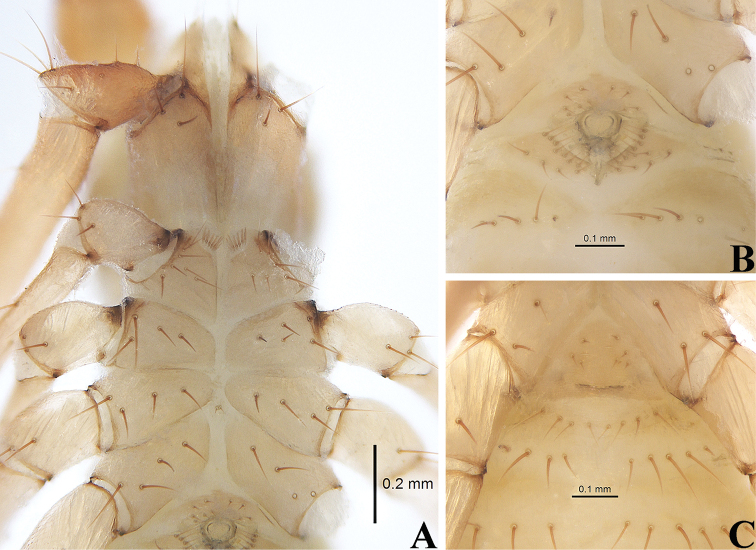
*Spelaeochthoniuswulibeiensis* sp. nov., holotype male (**A, B**), paratype female (**C**) **A** coxae (ventral view) **B** male genital area (ventral view) **C** female genital area (ventral view). Scale bars: 0.20 mm (**A**); 0.10 mm (**B, C**).

**Figure 22.**
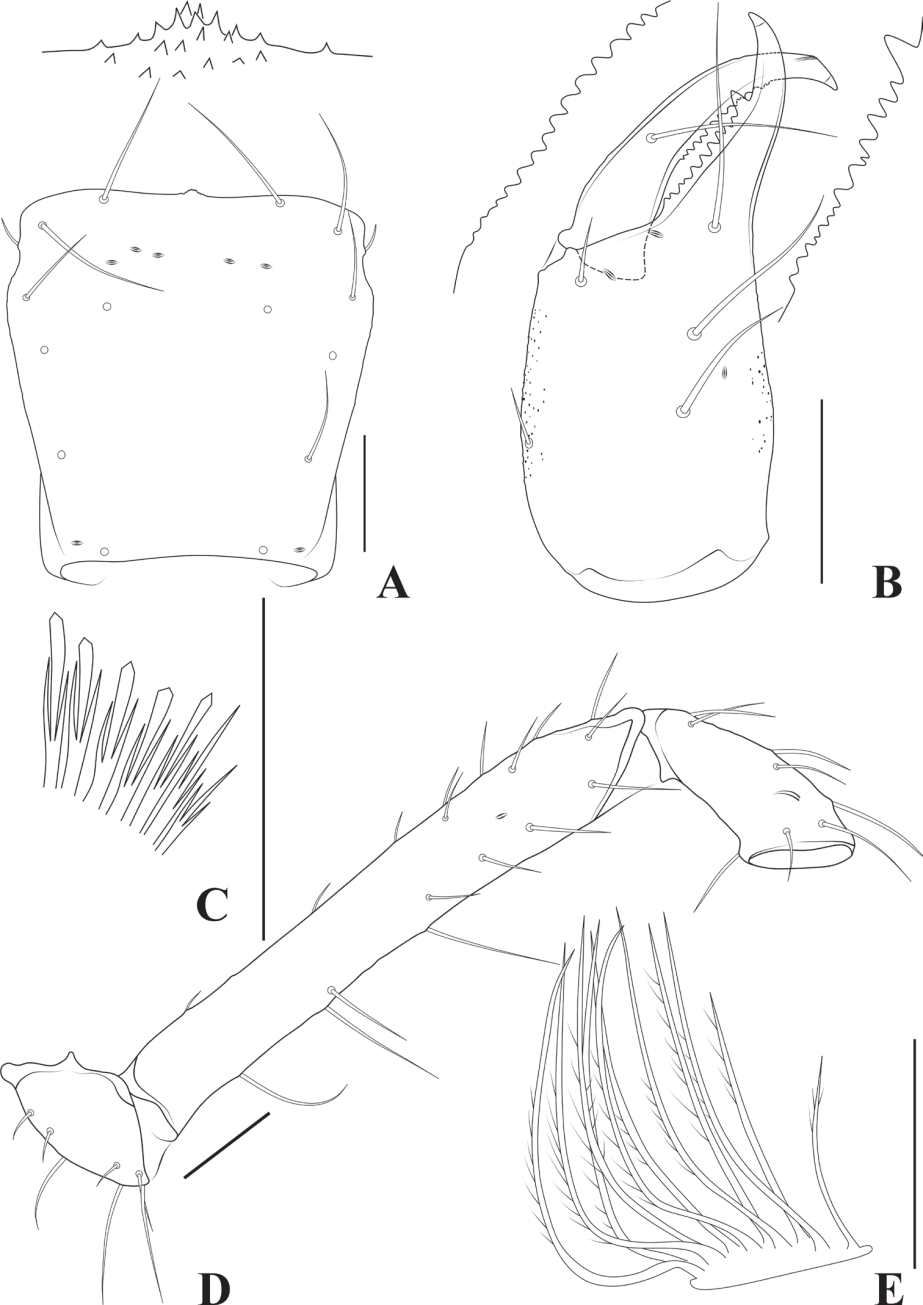
*Spelaeochthoniuswulibeiensis* sp. nov., holotype male **A** carapace (dorsal view), with a detail of anterior margin **B** left chelicera (dorsal view), with details of teeth **C** coxal spines on coxae I (ventral view) **D** left pedipalp (minus chela, dorsal view) **E** rallum. Scale bars: 0.20 mm (**A, B, D**); 0.10 mm (**C, E**).

**Figure 23.**
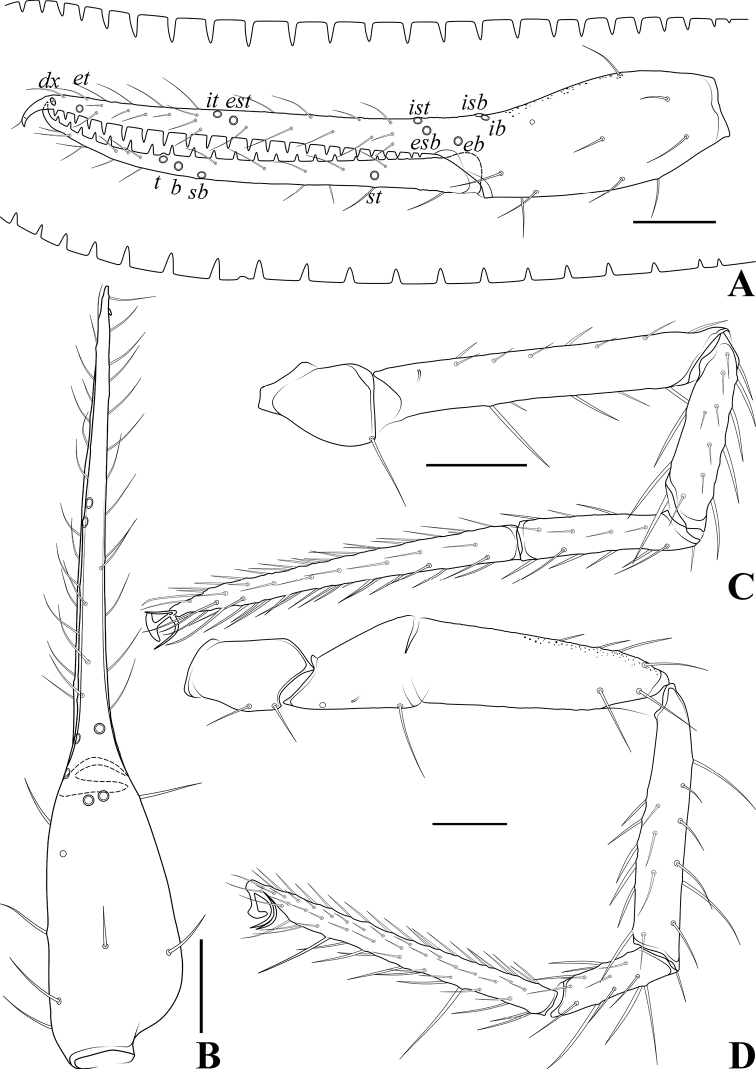
*Spelaeochthoniuswulibeiensis* sp. nov., holotype male **A** left chela (lateral view), with details of teeth and trichobothrial pattern **B** left chela (dorsal view) **C** leg I (lateral view) **D** leg IV (lateral view). Scale bars: 0.20 mm.

Figs 1A , 18 , 19 , 20 , 21 , 22 , 23 Chinese name 五里碑穴伪蝎

##### Type material.

***Holotype***: China • ♂; Guizhou Province, Weining County, Yancang Town, Yangguan Village, Wulibei Cave; 26°53.82'N, 104°19.36'E; 2425 m a.s.l.; 07 Aug. 2019; Zegang Feng, Zhaoyi Li and Chen Zhang leg.; under a stone in the deep zone; Ps.-MHBU-GZC19080701 (Figs 1A, 18). ***Paratypes***: • 2♂; the same data as the holotype; Ps.-MHBU-GZC19080702-GZC19080703 • 1♀; the same location as the holotype; 19 May. 2017, Zhisheng Zhang, Huiming Chen and Luyu Wang leg.; Ps.-MSWU-CZCH-17-06.

##### Diagnosis

**(♂♀).** The new species can be recognized by the following combination of characters: surfaces mostly with fine reticulations; carapace without eyes or eyespots but eye region bulging and convex in dorsal view; anterior margin without protuberances; cheliceral palm with five setae; rallum with 11 blades (each with fine pinnate, the basal-most blade shorter than the others); coxal spines present on coxa I only, comprising a transverse, contiguous series of seven or eight tridentate blades, which arise from a lightly sclerotized or translucent hillock, the central ramus of each blade (except the basal two) sharply acumino-spatulate and extending beyond the lateral rami; pedipalps slender, femur 7.24 (♂), 6.40 (♀), chela 6.21–6.22 (♂), 5.68 (♀) × longer than broad, both chelal fingers with a row of teeth (fixed chelal finger with 22 or 24 teeth; movable chelal finger with 16–19 teeth), slightly retrorse and pointed; chela fingers straight in dorsal view.

##### Etymology.

Named after the type locality, Wulibei Cave.

##### Description.

**Adult males** (Figs 18D, 19A, 20, 21A, B, 22, 23). ***Color*** (Figs 18D, 19A, 20, 21A, B): generally pale yellow, chelicerae, pedipalps and tergites slightly darker, soft parts pale. ***Cephalothorax*** (Figs 20B, 21A, 22A, C): carapace subquadrate, 1.02–1.03× longer than broad, gently narrowed posteriorly; surface mostly with fine reticulations, without furrows but with seven or eight lyrifissures; no traces of eyes but eye region bulging and convex in dorsal view; epistome present and with some tiny spinules; with 16 setae arranged s4s: 4: 2: 2: 2, most setae heavy, long, and gently curved. Chaetotaxy of coxae: P 3, I 6–7, II 4–5, III 4, IV 4; manducatory process with two acuminate distal setae, anterior seta less than 1/2 length of medial seta; coxal spines present on coxa I only, comprising a transverse, contiguous series of seven or eight tridentate blades, which arise from a lightly sclerotized or translucent hillock, the central ramus of each blade (except the basal two) sharply acumino-spatulate and extending beyond the lateral rami (Figs 21A, 22C); bisetose intercoxal tubercle present between coxae III and IV, tear drop-shaped (Fig. 21A). ***Chelicera*** (Figs 20C, 22B, E): large, approximately as long as carapace, 2.37–2.41× longer than broad; five setae present on hand, movable finger with a medial seta, all setae acuminate, ventrobasal seta shorter than others; exterior condylar lyrifissure and exterior lyrifissure exist, palm with one extra (between sub-basal seta and an accessory seta). Cheliceral palm with moderate hispid granulation on both ventral and dorsal sides. Both fingers well provided with teeth, fixed finger with 13–15 acute teeth, distal one largest; movable finger with 12 retrorse contiguous teeth of equal length, plus three or four round proximal teeth, 15 or 16 in total; galea represented by a very slight bump on movable finger. Serrula exterior with 21 blades and serrula interior with 17–20 blades. Rallum in two rows and composed of 11 blades with fine pinnate, of which the basal-most blade shorter than the others (Fig. 22E). ***Pedipalp*** (Figs 20A, 22D, 23A, B): surfaces mostly with fine reticulations; long and slender, trochanter 1.78–2.00, femur 7.24, patella 2.44–2.47, chela 6.21–6.22, hand 2.26–2.36× longer than broad; femur 2.62–2.80× longer than patella; movable chelal finger 1.61–1.74× longer than hand and 0.61–0.63× longer than chela. Setae generally long and acuminate; one distal lyrifissure present on patella and femur, respectively (Fig. 22D). Chelal palm robust and slightly constricted towards fingers. Fixed chelal finger and hand with eight trichobothria, movable chelal finger with four trichobothria, *ib*, *isb*, *eb*, *esb*, and *ist* clustered at the base of fixed finger, *ist* slightly distal to *esb*, *esb* close to *ist* than to *eb*; *it* slightly distal to *est*, situated subdistally and forming a pair; *et* situated subdistally, very close to chelal teeth; *dx* situated distal to *et*, near the tip of fixed finger; *sb* distinctly closer to *b* than to *st* (Fig. 23A). Microsetae (chemosensory setae) absent on hand and both palpal fingers. Sensilla absent. Both chelal fingers with a row of teeth, homodentate, spaced regularly along the margin, larger and well-spaced teeth present in the middle of the row, becoming smaller and closer distally and proximally: fixed chelal finger with 22–24 teeth, slightly retrorse and pointed; movable chelal finger with 16–19 teeth (slightly smaller than teeth on fixed chelal finger); a small tubercle between the seventh and eighth teeth present (near trichobothrium *t*) (Fig. 23A). Chelal fingers straight in dorsal view (Fig. 23B). ***Opisthosoma***: generally typical, ovate, pleural membrane finely granulated. Tergites and sternites undivided; setae uniseriate and acuminate. Tergal chaetotaxy I–XII: 2: 4: 6: 6: 6: 7–8: 8: 7: 6: 4: TT: 0, tergites VII–IX each with an unpaired median seta, one lyrifissure present on each side of tergites IV–IX. Sternal chaetotaxy III–XII: 6–9: 10–14: 13–14: 12: 12–13: 12–13: 10–11: 8–9: 0: 2, one lyrifissure present on each side of tergite III. Anterior genital operculum with 10–12 setae, genital opening pit-like, with seven marginal setae on each side, 24–26 in total, with a pair of lyrifissures present anterolateral and posteriolateral to genital opening, respectively (Fig. 21B). ***Legs*** (Fig. 23C, D): generally typical, long, and slender. Fine granulation present on anterodorsal faces of femur IV and patella IV. Femur of leg I 1.74× longer than patella and with one lyrifissure at the base of femur; tarsus 2.11–2.26× longer than tibia. Femoropatella of leg IV 3.70–3.88× longer than deep and with one lyrifissure at the base of femur; tibia 5.92–6.00× longer than deep; with a long tactile seta on both tarsal segments: basitarsus 4.00× longer than deep (TS = 0.22–0.28), telotarsus 11.13–12.43× longer than deep and 2.42–2.47× longer than basitarsus (TS = 0.26–0.27). Setae of leg I (trochanter to tibia) 1: 13: 12–15: 14–17, setae of leg IV (trochanter to basitarsus) 2: 2: 6: 16: 14. Arolium slightly shorter than the claws, not divided; claws simple. ***Dimensions of adult males*** (length/breadth or, in the case of the legs, length/depth in mm). Males: body length 2.50. Pedipalps: trochanter 0.32/0.16–0.18, femur 1.23/0.17, patella 0.44–0.47/0.18–0.19, chela 1.68–1.74/0.27–0.28, hand 0.61–0.66/0.27–0.28, movable finger length 1.06. Chelicera 0.64–0.65/0.27, movable finger length 0.34. Carapace 0.63/0.61–0.62. Leg I: trochanter 0.24–0.25/0.18, femur 0.73–0.75/0.10, patella 0.42–0.43/0.09, tibia 0.34–0.37/0.07, tarsus 0.77–0.78/0.06–0.07. Leg IV: trochanter 0.33–0.36/0.19–0.22, femoropatella 0.97–1.00/0.25–0.27, tibia 0.77–0.78/0.13, basitarsus 0.36/0.09, telotarsus 0.87–0.89/0.07–0.08.

**Adult female** (Figs 19B, 21C). Mostly same as males; tergal chaetotaxy I–XII: 2: 4: 6: 6: 6: 7: 8: 9: 7: 4: TT: 0; sternal chaetotaxy IV–XII: 10: 13: 11: 12: 12: 11: 8: 0: 2; anterior genital operculum with seven setae, posterior margin with 11 marginal setae, 18 in total; leg IV with a long tactile seta on both tarsal segments: basitarsus 3.60× longer than deep (TS = 0.25), telotarsus 11.63× longer than deep and 2.58× longer than basitarsus (TS = 0.24). Body length 1.93. Pedipalps: trochanter 0.32/0.19 (1.68×), femur 1.28/0.20 (6.40×), patella 0.51/0.21 (2.43×), chela 1.76/0.31 (5.68×), hand 0.66/0.31 (2.13×), movable chelal finger length 1.11. Chelicera 0.70/0.30 (2.33×), movable finger length 0.38. Carapace 0.68/0.68 (1.00×). Leg I: trochanter 0.24/0.19 (1.26×), femur 0.77/0.12 (6.42×), patella 0.45/0.11 (4.09×), tibia 0.36/0.08 (4.50×), tarsus 0.83/0.08 (10.38×). Leg IV: trochanter 0.35/0.22 (1.59×), femoropatella 1.05/0.27 (3.89×), tibia 0.80/0.14 (5.71×), basitarsus 0.36/0.10 (3.60×), telotarsus 0.93/0.08 (11.63×).

##### Remarks.

The new species shares similar characters with most species of *Centrochthonius* Beier, 1931, *Spelaeochthonius* and all species of “*Pseudotyrannochthonius*” Beier, 1930 from the western US by the presence of only 16 setae on the carapace. Schwarze et al. (2021) emphasized the importance of the number of carapaceal setae in Holarctic pseudotyrannochthoniids, thus, it indicates that the three “*Pseudotyrannochthonius*” species in the western US were misclassified in comparison with the twelve species from Australia and the three species from Chile (including the type species, the number of carapaceal setae is more than 18). It can be said that the genus *Pseudotyrannochthonius* is endemic to the southern hemisphere (Harvey and Harms 2022). Thus, it is inappropriate to place this new species in *Pseudotyrannochthonius*, even though the shape of this new species of coxal spines is similar to that of the three “*Pseudotyrannochthonius*” species.

The shape and number of the coxal spines are important distinguishing features between *Centrochthonius* and *Spelaeochthonius* (You et al. 2022). In our opinion, it is appropriate to place this new species to *Spelaeochthonius* rather than *Centrochthonius*, the reasons are as follows: for *Centrochthonius*, the number of carapaceal setae is not fixed (e.g., occasionally 18 are present in *C.anatonus* Harvey & Harms, 2022) and only four or five coxal spines blades; for *Spelaeochthonius*, the character of coxal spines is diverse (e.g., in *S.undecimclavatus* Morikawa, 1956, which is club-shaped, not distally plumose).

*Spelaeochthoniuswulibeiensis* sp. nov. is similar to *S.cheonsooi* You, Yoo, Harvey & Harms, 2022, but differs by the number of setae on tergite I (2 vs. 4) and larger body size (body length 1.93 (♀) mm vs. 1.70 (♀) mm; chela 5.68 (♀) × vs. 5.32 (♀) × longer than board, length 1.76 (♀) mm vs. 1.49 (♀) mm).

*Spelaeochthoniuswulibeiensis* sp. nov. can be distinguished from *S.seungsookae* You, Yoo, Harvey & Harms, 2022 by the number of setae on tergite I (2 vs. 4) and smaller body size (body length 1.93 (♀) mm vs. 2.05–2.36 (♀) mm; chela length 1.68–1.74 (♂), 1.76 (♀) mm vs. 1.90 (♂), 1.92 (♀) mm); from *S.undecimclavatus* and *S.dorogawaensis* by the number of setae on chelicera (6 vs. 7), a slender palp (palpal femur 7.24 (♂) × vs. 4.80–5.40 (♂) × longer than board; chela 6.21–6.22 (♂) × vs. 5.50–6.13 (♂) × longer than board) and lower number of blades of coxal spines (7–8 vs. 10–11); from *S.akiyoshiensis* Morikawa, 1956 and *S.kobayashii* Morikawa, 1956 by the number of setae on chelicera (6 vs. 7), lower number of movable chelal finger teeth (16–19 teeth vs. min. 26 teeth) and a slender palp (palpal femur 7.24 (♂) × vs. 5.00–5.60 (♂) × longer than board; chela 6.21–6.22 (♂) × vs. 5.13–5.74 (♂) × longer than board); from *S.dentifer* (Morikawa, 1970) by the number of setae on chelicera (6 vs. 7), lower number of movable chelal finger teeth (16–19 teeth vs. min. 36 teeth) and a shorter chela (chela 6.21–6.22 (♂), 5.68 (♀) × vs. 6.85 (♂), 7.12 (♀) × longer than board, length 1.68–1.74 (♂), 1.76 (♀) mm vs. 1.85 (♂♀) mm); from *S.kubotai* by the slightly smaller body size (body length 1.93 (♀) mm vs. 2.03 (♀) mm; chela 5.68 (♀) × vs. 5.70 (♀) × longer than board; movable chelal finger 1.68 (♀) × vs. 1.87 (♀) × longer than) and the number of setae of coxal spines (7 or 8 vs. 11); from *S.kishidai* (Morikawa, 1960) by a slender palp (palpal femur 7.24× vs. 4.90× longer than board; movable chelal finger 0.61–0.63× vs. 0.67–0.69× longer than board) (Morikawa 1954, 1956, 1960, 1970; You et al. 2022).

##### Distribution and habitat.

This species is known only from the type locality, Wulibei Cave (Figs 1A, 18A–C), which is located ~ 1.2 km east of Yangguan Village (Weining County). This limestone cave has an elongated entrance (~ 2.5 m high and 8 m wide) with some corn stalks scattered nearby. Entrance of the cave has a large muddy cave hall, connected to a small hall through a narrow tunnel, which is a more enclosed, completely dark space, covered with gravel, with temperatures ~ 10 °C and humidity ~ 90%. The specimen was collected under a stone in a small cave hall.

## Supplementary Material

XML Treatment for Allochthonius

XML Treatment for Allochthonius bainiensis

XML Treatment for Allochthonius pandus

XML Treatment for Allochthonius xinqiaoensis

XML Treatment for Spelaeochthonius

XML Treatment for Spelaeochthonius wulibeiensis

## References

[B1] Chamberlin JC (1931) The arachnid order Chelonethida. Stanford University Publications, University Series, [Biol. Sci.] 7(1): 1–284.

[B2] Feng ZG Wynne JJ Zhang F (2019) Two new subterranean-adapted pseudoscorpions (Pseudoscorpiones: Neobisiidae: Parobisium) from Beijing, China. Zootaxa 4661(1): 145–160. 10.11646/zootaxa.4661.1.7 31716721

[B3] Feng ZG Wynne JJ Zhang F (2020) Cave-dwelling pseudoscorpions of China with descriptions of four new hypogean species of *Parobisium* (Pseudoscorpiones, Neobisiidae) from Guizhou Province. Subterranean Biology 34: 61–98. 10.3897/subtbiol.34.49586

[B4] Gao ZZ Zhang YF Zhang F (2016) Two new species of Pseudotyrannochthoniidae, including the first species of *Pseudotyrannochthonius* (Pseudoscorpiones) from China. Acta Zoologica Academiae Scientiarum Hungaricae 62(2): 117–131. 10.17109/AZH.62.2.117.2016

[B5] Gao ZZ Chen HM Zhang F (2017) Description of two new cave-dwelling *Bisetocreagris* species (Pseudoscorpiones: Neobisiidae) from China. Turkish Journal of Zoology 41: 615–623. 10.3906/zoo-1602-39

[B6] Gao ZZ Wynne JJ Zhang F (2018) Two new species of cave-adapted pseudoscorpions (Pseudoscorpiones: Neobisiidae, Chthoniidae) from Guangxi, China. The Journal of Arachnology 46(2): 345–354. 10.1636/JoA-S-17-063.1

[B7] Gao ZZ Zhang F Chen HM (2020) Two new cave-dwelling species of *Tyrannochthonius* Chamberlin, 1929 (Pseudoscorpiones: Chthoniidae) from the Guizhou karst, China. Zootaxa 4853(4): 572–580. 10.11646/zootaxa.4853.4.6 33056361

[B8] Harvey MS (1992) The phylogeny and classification of the Pseudoscorpionida (Chelicerata: Arachnida). Invertebrate Taxonomy 6(6): 1373–1435. 10.1071/IT9921373

[B9] Harvey MS Harms D (2022) The pseudoscorpion genus *Centrochthonius* (Pseudoscorpiones: Pseudotyrannochthoniidae) from central Asia and description of a new species from Nepal. The Journal of Arachnology 50(2): 158–174. 10.1636/JoA-S-21-033

[B10] Hou YM Gao ZZ Zhang F (2022a) Two new species of cave-adapted pseudoscorpions (Pseudoscorpiones, Chthoniidae) from Yunnan, China. ZooKeys 1097: 65–83. 10.3897/zookeys.1097.82527 35837584 PMC9046370

[B11] Hou YM Gao ZZ Zhang F (2022b) Diversity of cave-dwelling pseudoscorpions from eastern Yunnan in China, with the description of eleven new species of the genus *Lagynochthonius* (Pseudoscorpiones, Chthoniidae). Zootaxa 5198(1): 001–065. 10.11646/zootaxa.5198.1.1 37045058

[B12] Hu JF Zhang F (2011) Description of three new species of the genus *Allochthonius* Chamberlin, 1929 (Pseudoscorpiones: Pseudotyrannochthoniidae) from China. Journal of Threatened Taxa 3(11): 2167–2176. 10.11609/JoTT.o2767.2167-76

[B13] Hu JF Zhang F (2012) Two new species of the genus *Allochthonius* Chamberlin from China (Pseudoscorpiones: Pseudotyrannochthoniidae). Entomologica Fennica 22(4): 243–248. 10.33338/ef.5003

[B14] Judson MLI (2007) A new and endangered species of the pseudoscorpion genus *Lagynochthonius* from a cave in Vietnam, with notes on chelal morphology and the composition of the Tyrannochthoniini (Arachnida, Chelonethi, Chthoniidae). Zootaxa 1627(1): 53–68. 10.11646/zootaxa.1627.1.4

[B15] Latella L (2019) Biodiversity: China. In: White WB Culver DC Pipan T (Eds) Encyclopedia of Caves (3rd Edn).Academic Press, Amsterdam, 127–135. 10.1016/B978-0-12-814124-3.00016-9

[B16] Li YC (2022) Five new troglobitic species of *Tyrannochthonius* (Arachnida, Pseudoscorpiones, Chthoniidae) from the Yunnan, Guizhou and Sichuan Provinces, China. ZooKeys 1131: 173–195. 10.3897/zookeys.1131.91235 PMC9836568 36761461

[B17] Li YC Wang ML (2021) Description of a new cave-dwelling pseudoscorpion, *Anatemnushemuensis* sp. nov. (Pseudoscorpiones, Atemnidae) from Gaoligong Mountain, China. Carsologica Sinica 40(6): 1058–1062.

[B18] Li YC Shi AM Liu H (2017) A new cave-dwelling species of *Bisetocreagris* Ćurčić (Arachnida, Pseudoscorpiones: Neobisiidae) from Yunnan Province, China. Entomologica Fennica 28(4): 212–218. 10.33338/ef.84688

[B19] Li YC Liu H Shi AM (2019) A new cave-dwelling species of *Lagynochthonius* (Arachnida: Pseudoscorpiones: Chthoniidae) from Yunnan Province, China. Zootaxa 4571(1): 28–34. 10.11646/zootaxa.4571.1.2 31715828

[B20] Lin JQ (2001) The distributed area and the features analysis of karst and non karst landscape in Guizhou. Journal of Guizhou Educational College 04: 43–46.

[B21] Mahnert V (2003) Four new species of pseudoscorpions (Arachnida, Pseudoscorpiones: Neobisiidae, Chernetidae) from caves in Yunnan Province, China. Revue Suisse de Zoologie 110: 739–748. 10.5962/bhl.part.80209

[B22] Mahnert V (2009) New species of pseudoscorpions (Arachnida, Pseudoscorpiones, Chthoniidae, Chernetidae) from caves in China. Revue Suisse de Zoologie 116(2): 185–201. 10.5962/bhl.part.79492

[B23] Mahnert V Li YC (2016) Cave-inhabiting Neobisiidae (Arachnida: Pseudoscorpiones) from China, with description of four new species of Bisetocreagris Ćurčić. Revue Suisse de Zoologie 123: 259–268.

[B24] Morikawa K (1954) On some pseudoscorpions in Japanese lime-grottoes. Memoirs of Ehime University 2(2B): 79–87.

[B25] Morikawa K (1956) Cave pseudoscorpions of Japan (I). Memoirs of Ehime University 2(2B): 271–282.

[B26] Morikawa K (1960) Systematics studies of Japanese pseudoscorpions. Memoirs of Ehime University 2(2B): 85–172.

[B27] Morikawa K (1970) Results of the speleological survey in South Korea 1966. XX. New pseudoscorpions from South Korea. Bulletin of the National Science Museum, Tokyo 13: 141–148.

[B28] Schawaller W (1995) Review of the pseudoscorpion fauna of China (Arachnida: Pseudoscorpionida). Revue Suisse de Zoologie 102(4): 1045–1063. 10.5962/bhl.part.80489

[B29] Schwarze D Harms D Hammel J Kotthoff U (2021) The first fossils of the most basal pseudoscorpion family (Arachnida: Pseudoscorpiones: Pseudotyrannochthoniidae): Evidence for major biogeographical shifts in the European paleofauna. PalZ 96(1): 11–27. 10.1007/s12542-021-00565-8

[B30] Viana ACM Ferreira RL (2021) A new troglobitic species of Allochthonius (subgenus Urochthonius) (Pseudoscorpiones, Pseudotyrannochthoniidae) from Japan. Subterranean Biology 37: 47–55. 10.3897/subtbiol.37.58580

[B31] WPC (2022) World Pseudoscorpiones Catalog. Natural History Museum Bern. https://wac.nmbe.ch/order/pseudoscorpiones/3 [Accessed on 30 Nov. 2022]

[B32] Xu HR Gao ZZ Zhang F (2022) Two new species of the pseudoscorpion subfamily Lamprochernetinae Beier, 1932 from Guizhou, China (Pseudoscorpiones: Chernetidae). Zootaxa 5105(4): 581–592. 10.11646/zootaxa.5105.4.7 35391285

[B33] You J Yoo JS Harvey MS Harms D (2022) Some cryptic Korean karst creatures: revalidation of the pseudoscorpion genus *Spelaeochthonius* (Pseudoscorpiones: Pseudotyrannochthoniidae) and description of two new species from Korea. The Journal of Arachnology 50(2): 135–157. 10.1636/JoA-S-21-025

[B34] Zhang FB Zhang F (2014) A new species of the genus *Allochthonius* (Pseudoscorpiones: Pseudotyrannochthoniidae) from Liupan mountains, China, with description of the male of *Allochthoniusbrevitus*. Acta Zoologica Academiae Scientiarum Hungaricae 60(1): 45–56.

[B35] Zhang DF Ouyang ZY Wang SJ (2001) Population resources environment and sustainable development in the karst region of Southwest China. Zhongguo Renkou Ziyuan Yu Huanjing 11(1): 77–81. [In Chinese]

[B36] Zhang C Feng ZG Zhang F (2020) Two new cave-dwelling pseudoscorpions (Pseudoscorpiones: Neobisiidae: Parobisium) from Yunnan, China. Zootaxa 4834(1): 107–120. 10.11646/zootaxa.4834.1.7 33056135

